# Label-free integrative pharmacology on-target of opioid ligands at the opioid receptor family

**DOI:** 10.1186/2050-6511-14-17

**Published:** 2013-03-12

**Authors:** Megan Morse, Haiyan Sun, Elizabeth Tran, Robert Levenson, Ye Fang

**Affiliations:** 1 Department of Pharmacology, Pennsylvania State University College of Medicine, Hershey, PA, USA; 2 Biochemical Technologies, Science and Technology Division, Corning Inc., Corning, NY, USA

**Keywords:** Opioid receptor, Functional selectivity, Label-free biosensor

## Abstract

**Background:**

*In vitro* pharmacology of ligands is typically assessed using a variety of molecular assays based on predetermined molecular events in living cells. Many ligands including opioid ligands pose the ability to bind more than one receptor, and can also provide distinct operational bias to activate a specific receptor. Generating an integrative overview of the binding and functional selectivity of ligands for a receptor family is a critical but difficult step in drug discovery and development. Here we applied a newly developed label-free integrative pharmacology on-target (iPOT) approach to systematically survey the selectivity of a library of fifty-five opioid ligands against the opioid receptor family. All ligands were interrogated using dynamic mass redistribution (DMR) assays in both recombinant and native cell lines that express specific opioid receptor(s). The cells were modified with a set of probe molecules to manifest the binding and functional selectivity of ligands. DMR profiles were collected and translated to numerical coordinates that was subject to similarity analysis. A specific set of opioid ligands were then selected for quantitative pharmacology determination.

**Results:**

Results showed that among fifty-five opioid ligands examined most ligands displayed agonist activity in at least one opioid receptor expressing cell line under different conditions. Further, many ligands exhibited pathway biased agonism.

**Conclusion:**

We demonstrate that the iPOT effectively sorts the ligands into distinct clusters based on their binding and functional selectivity at the opioid receptor family.

## Background

Historically, drug selectivity is described as the differential binding affinity of drug molecules to distinct receptors. The discovery of ligand-directed functional selectivity or biased agonism has led to new avenues for achieving desired drug selectivity. Functional selectivity describes the differential ability of drug molecules to activate one of the multiple downstream pathways to which the receptor is coupled [1-4]. Opioid receptors exemplify many aspects of functional selectivity, with the dependency of receptor-mediated events on ligands used and the cellular or *in vivo* environments examined [5]. Functional selectivity of opioid drugs has been postulated to be related to their clinical profiles, particularly the progression of analgesic tolerance after their extended use [6].

However, integrating functional selectivity into the drug development process remains a challenging problem. The wide spectrum of signaling events mediated by a receptor [7], coupled with the differences in signaling components in distinct types of cells [8], makes it extremely difficult to fully discover and quantify the functional selectivity of drug molecules using conventional molecular assays. Also, these molecular assays screen drug molecules based on a predetermined molecular hypothesis, but such a hypothesis may or may not be relevant to the pathogenesis of a disease [9]. A further complication is the existence of signaling readout- and cell background-dependent potency and efficacy, which is inherited from the operational bias of drug molecules on a receptor [3]. The possibility that a drug may have multidimensional efficacy makes it difficult to optimize and prioritize drug candidate molecules. In many instances, the efficacy profiles obtained for a candidate drug may not be good predictors of their *in vivo* therapeutic impacts, and it may be difficult to sort out which molecular mode of action leads to a desired therapeutic impact. Thus, assays that are phenotypic in nature yet allow mechanistic descriptions of drug actions would be advantageous.

With the ability to interrogate wide pathway coverage utilizing a single assay and to mechanistically delineate drug pharmacology at the whole cell or cell system level, label-free receptor assays have emerged as promising platforms for drug discovery [10-14]. Here, we applied a recently developed label-free integrative pharmacology on-target (iPOT) approach [15,16] to systematically survey the binding and functional selectivity of a library of opioid ligands. This comparative pharmacological approach is centered on similarity analysis of DMR profiles of drugs obtained in model cell lines that have been pretreated with a wide variety of probe chemicals. The probe molecules are chosen to modify pathways downstream of activated receptors, so that the sensitivity of drugs to the pathway modulation can be surveyed at the whole cell level. After translating DMR profiles into multidimensional coordinates, similarity analysis is used to categorize drugs into distinct clusters. We found that the iPOT approach provides an integrative display of the binding and functional selectivity of a library of opioid ligands at the family of opioid receptors.

## Methods

### Materials and reagents

Pertussis toxin (PTX), cholera toxin (CTX), forskolin and dimethyl sulfoxide (DMSO) were purchased from Sigma-Aldrich (St. Louis, MO). DAMGO, DPDPE, BRL-52537, CTOP, naltrindole hydrochloride, norbinaltorphimine, U0126, SB202190, SP600125, and LY294002 were purchased from Tocris Biosciences (Ellisville, MO). The Opioid Compound Library consisting of 64 compounds of pan-specific and receptor subtype-specific agonists and antagonists, each at 10 mM in DMSO, was obtained from Enzo Life Sciences (Plymouth Meeting, PA). All tissue culture media and reagents were purchased from Invitrogen (Calrsbad, CA). Both fibronectin-coated and tissue culture treated (TCT) Epic® biosensor microplates, as well as polypropylene compound source plates were obtained from Corning Inc (Corning, NY).

### Cell culture

We used five distinct cell lines including human neuroblastoma cell line SH-SY5Y, human embryonic kidney HEK293 cells, and three engineered HEK 293 cell lines for label-free pharmacology profiling. HEK293 cells and SH-SY5Y cells were obtained from American Type Tissue Culture (Manassas, VA) and cultured in Dulbecco’s modified Eagle’s medium (DMEM GlutaMAX-I) supplemented with 10% non-heated inactivated fetal bovine serum, 100 units/ml penicillin, and 100 g/ml streptomycin. Both HEK-MOR and HEK-DOR cell lines were a generous gift from Dr. Mark von Zastrow (University of California, San Francisco). The HEK-KOR cell line was donated from Dr. Lee-Yuen Lui-Chen (Temple University).

The HEK-MOR stably expresses FLAG-tagged wild type human mu opioid receptor (MOR1) with a Bmax of 2.5 pmoles/mg cell protein [16,17]. The HEK-DOR stably expresses FLAG-tagged wild type human delta opioid receptor with a Bmax of 0.8 pmoles/mg cell protein [18]. The HEK-KOR cell line stably expresses FLAG-tagged wild type human kappa opioid receptor with an unknown Bmax [19]. SH-SY5Y is a dopaminergic neuronal cell line which has been used as an *in vitro* model for assessment of functional responses of the MOR. SH-SY5Y is known to express both MOR and DOR with a protein ratio of approximately 4.5:1 [20], and the Bmax for the DOR was estimated to be 35 to 100 fmol/mg protein [21,22]. SH-SH5Y is also known to endogenously express several splice variants of opioid receptors including a single TM protein (MOR1S) resulting from an exon-skipping variant [23,24], an alternatively spliced isoform MOR1K that is a 6TM GPCR variant without the N-terminal extracellular and first transmembrane domains and is preferentially coupled to G_αs_[25], and a splice variant of δ opioid receptor that lacks the third cytoplasmic loop of the native receptor [26]. This short δ receptor appeared to be associated with human malignoma, although its biological functions remain unknown.

These cells were grown in complete DMEM GlutaMAX-I containing 400 μg/ml geneticin. For cell culture in the fibronectin-coated Epic® biosensor microplates, cells were seeded at a density of 16,000 cells/40 μL/well for HEK293 cells, and 20,000 cells/40 μL/well for both HEK-DOR and HEK-KOR cells. For SH-SY5Y cells, cells were seeded at 15,000 cells/40 μL/well onto Epic® tissue culture compatible microplates. After seeding the biosensor microplates were incubated for 30 min at room temperature, and then transferred to a humidified incubator (37°C, 5% CO_2_) for 20–24 hrs for HEK cells, or 48 hours for SH-SY5Y cells.

### Dynamic mass redistribution (DMR) assays

DMR assays were performed using Epic® system as previously described [27]. Epic® system from Corning is a wavelength interrogation reader system tailored for resonant waveguide grating biosensors in microplates. This system consists of a temperature-control unit (26°C), an optical detection unit, and an on-board liquid handling unit with robotics. The detection unit is centered on integrated fiber optics, and enables kinetic measures of cellular responses with a time interval of ~15 sec.

For DMR assays, once reached high confluency (~95%) the cells were washed twice with assay buffer (1× Hank’s balanced salt solution with 20 mM HEPES, pH7.1) and transferred to the Epic® reader for 1 hr at 26°C so a steady baseline was reached. DMR was monitored in real time with a temporal resolution of ~15 sec throughout the assays. A typical DMR proceeded with a 2-min baseline, followed by a real time kinetic response after the compound additions using the onboard liquid handler. The DMR was recorded as a shift in resonant wavelength (picometer, pm). Different DMR assay formats were used for profiling opioid ligands. DMR agonist assays were used to directly record the DMR signal arising from a ligand itself. DMR antagonist assays were used to record the DMR arising from an agonist at a fixed dose (usually its EC_100_) after pretreatment with an inhibitor or a ligand. An EC_100_ value was used to ensure maximal activation of respective receptor for follow-up potency studies in order to have greater antagonism differentiation power than the dose at its EC_50_.

For iPOT profiling, all ligands were examined at 10 μM. This was based on three obvious reasons. First, different ligands often display a wide range of affinities binding to a specific receptor, and a specific ligand often displays distinct affinities binding to different opioid receptors (Additional file 1). Furthermore, the binding affinity of a ligand often does not directly translate to its potency to activate the receptor at the whole cell level [27,28], so it is practically difficult to choose ligand-specific concentrations for our systematic profiling. Second, the main purpose of the present study is to determine both binding and functional selectivity of the same family of ligands against the opioid receptor family, and almost all ligands examined displayed agonist activity in at least one of the five cell lines profiled (see results below). Thus, it is necessary to use a high concentration to saturate the receptor sites and to maximize the functional activation of the receptors induced by most, if not all, of the ligands examined. Third, 10 μM is the most commonly used concentration for high throughput screening and profiling.

To manifest the specificity, relative potency and efficacy, and modes of action of the drugs, a variety of probe molecules were used to achieve a wide range of chemical environments for each cell line through alteration of cellular signaling protein(s) in the signaling pathways of opioid receptors. Here, the cells were pretreated offline with several probe molecules by incubating the cells with a probe molecule at the indicated dose for the indicated period of time (Table 1). After the pretreatment with the probe molecules the cells were then stimulated with an opioid ligand, whose responses were recorded in real time and used for similarity and correlation analysis. Specifically, cells were pretreated with either 0.1% DMSO (the positive control), 10 μM opioid ligand in the library, 100 ng/ml PTx, 400 ng/ml CTX, 10 μM forskolin, 10 μM U0126, 10 μM SB202190, 10 μM SP600125, or 10 μM LY294002 for the times indicated. Since the primary purpose of the iPOT profiling of opioid ligands was to identify interesting ligands for quantitative pharmacology assessment, all kinase inhibitors at 10 μM were used to manifest the sensitivity of the label-free profiles of opioid ligands to the pathway modulation. PTX binds to G_αi_, resulting in inhibition of G_αi_ by ADP ribosylation of a Cys residue and uncoupling of the G protein from the receptor [29]. CTX binds to G_αs_, resulting in activation of G_αs_ by ADP ribosylation of an Arg residue and cAMP production [30]. Forskolin is an activator of adenylyl cyclase and is widely used for cell-based screening due to its ability to increase G_αi_-mediated signaling but desensitize G_αs_-mediated signaling [31]. U0126, SB202190, SP600125, and LY294002 are known kinase pathway inhibitors for MEK1/2, p38 MAPK, JNK, and PI3K, respectively [32,33]. It has been suggested that opioid ligands often exhibit functional selectivity on these pathways [5,6,34]. It is worthy of noting that the results obtained using this approach may not directly translate into a pathway-specific biased agonism, given that many, if not all kinase inhibitors, display polypharmacology (that is, the ability to bind to more than one targets).

**Table 1:** Assay protocols and DMR signals used for similarity analysis

**Cell**	**Probe, pretreatment duration**	**DMR readout**	**Labels used in clustering**	**Figures**
HEK293	0.1% DMSO in buffer, 1 hr	Ligand, 10 μM	HEK-3, 9, 30	Additional file 1: Figure S1, S2
Opioid*	0.1% DMSO in buffer, 1hr	Ligand, 10 μM	Buffer-3, 9, 30	Figures 1,5,6,7
HEK-MOR	10 μM ligand, 1 hr	DAMGO, 10 μM	MOR-3, 9, 30	Additional file 1: Figures S2 & S3
HEK-DOR	10 μM ligand, 1 hr	DPDPE, 10 μM	DOR-3, 9, 30	Figure 5
HEK-KOR	10 μM ligand, 1 hr	BRL-57532, 10 μM	KOR-3, 9, 30	Figure 6
SH-SY5Y	10 μM ligand, 1 hr	DAMGO, 10 μM	5Y-3, 9, 30	Figure 7
Opioid*	100 ng/ml PTX, 20 hr	Ligand, 10 μM	PTX-3, 9, 30	Figures 5,6 and 7
Opioid*	400 ng/ml CTX, 20 hr	Ligand, 10 μM	CTX-3, 9, 30	Figures 5,6 and 7
Opioid*	10 μM forskolin, 1 hr	Ligand, 10 μM	FSK-3, 9, 30	Figures 5,6 and 7
Opioid*	10 μM U0126, 1 hr	Ligand, 10 μM	U0126-3, 9, 30	Figures 5,6 and 7
Opioid*	10 μM SB202190, 1 hr	Ligand, 10 μM	SB-3, 9, 30	Figures 5,6 and 7
Opioid*	10 μM SP600125, 1 hr	Ligand, 10 μM	SP-3, 9, 30	Figures 5,6 and 7
Opioid*	10 μM LY294002, 1 hr	Ligand, 10 μM	LY-3, 9, 30	Figures 5,6 and 7

* Opioid receptor expressing cell lines wherein the same assay protocol was applied.

We screened a library of 64 opioid ligands. Literature mining revealed that fifty-five of the opioid ligands in the library had previously been shown to possess binding affinity for at least one member of the classic opioid receptor family (Additional file 1: Tables S1–S3), and thus chosen for analysis in this study.

### Quantitative real-time PCR

Total RNA was extracted from SH-SY5Y or HEK293 cells using an RNeasy mini kit (Qiagen, Cat#74104). To eliminate genomic DNA contamination, on-column DNase digestion was performed using RNase-free DNase set from Qiagen (Valencia, CA). The concentration and quality of total RNA were determined using a Nanodrop 8000 from Thermo Scientific. Customized PCR-array plates for 352 GPCR genes and reagents were ordered from SABiosciences (Frederick, MD). About 1 μg total RNA was used for each 96-well PCR-array. The PCR-array was performed on an ABI 7300 Real-Time PCR System following the manufacturer’s instructions.

### Data visualization and clustering

**Figure 1:**
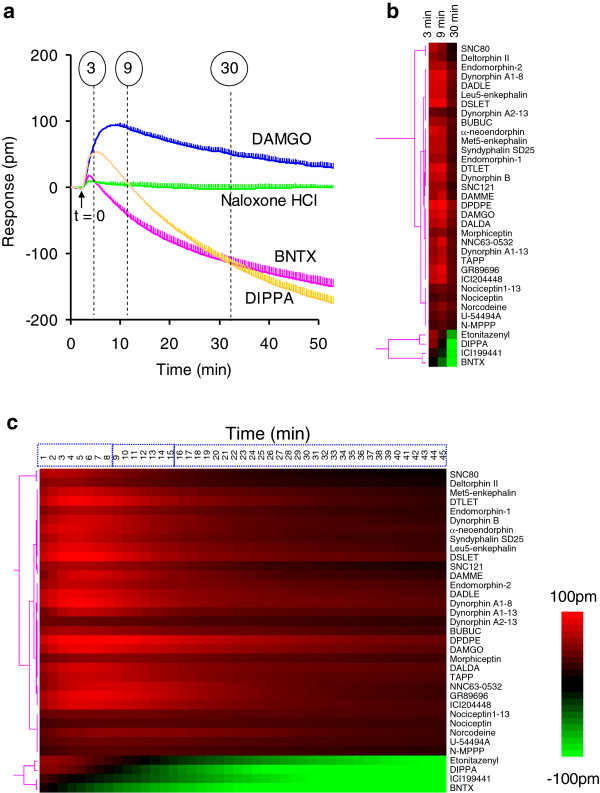
**Extracting DMR parameters for effective similarity analysis.** (**a**) Representative DMR signals of opioid ligands in native SH-SY5Y cells. The data represents the mean ± s.d. of 2 independent measurements, each in duplicate (n = 4). Responses at three time points (3 min, 9 min, and 30 min post-stimulation) were extracted to represent each DMR signal. The solid arrow indicates the time when ligands were added (t =0). (**b**) A colored heat map of opioid ligand-induced DMR in native SH-SY5Y cells based on the responses at the three time points. (**c**) A colored heat map based on the real time DMR signals of all opioid ligands that gave rise to a detectable DMR. The real time responses showed was reduced to every minute. Three time domains were evident.

For each opioid ligand in a cell line, ten DMR assays were performed that measured receptor specificity, G-protein coupling, and downstream kinase pathway selectivity. DMR assay offers a texture rich readout for ligand-receptor interactions at the whole cell level [11-13]. Originating from distinct functional selectivity and polypharmacology, the DMR signals of different ligands could be diverse in a specific cell [35]. To classify ligands, we adopt similarity analysis, a technology to cluster molecules through determination of the similarity and distances among a large set of different biological data [36-38]. For effective similarity analysis the real responses at three distinct time points (3 min, 9 min, and 30 min post-stimulation) were extracted from each kinetic DMR signal and used to rewrite the DMR pharmacology of each ligand. Combining DMR parameters from multiple assays and/or cells formed a numerical descriptor containing multi-dimensional coordinates for each ligand, which was then subject to similarity analysis. At least duplicate data for each assay were collected to generate an averaged response. For visualization, the real-time responses were color coded to illustrate relative differences in DMR signal strength. The red color refers to a positive value, the black a value near zero, and the green color represents a negative value. Differences in color intensity illustrate differences in signal strength. In the ligand matrix, each column represents one DMR response at a particular time under a specific assay condition, and each row represents one ligand. Every row and column carries equal weight. The Ward hierarchical clustering algorithm and Euclidean distance metrics [15,16] were used for generating heat maps and clustering the DMR profiles. To assist with direct visualization of DMR characteristics of each ligand in an assay, we did not carry out similarity analysis among distinct columns, except for the analysis based on real time responses (Figure 1). Each assay was arranged in three consecutive columns to form a column group for clear understanding of the key characteristics of a DMR.

### Statistical analysis

For profiling, two independent measurements, each done in duplicate, were performed. All replicates passed the 2 sigma coefficient of variation test in order to be included in the analysis. Drugs whose DMR responses failed the statistical test were re-screened. At least two replicates were included for the final analysis. For dose responses, at least two independent measurements, each done at least in duplicate, were performed to calculate the mean responses and the standard deviations (s.d.).

## Results

### Expression of endogenous opioid receptors

We performed quantitative real time PCR to determine the expression of endogenous opioid receptors in the parental HEK293 cell line as well as SH-SY5Y cells. Results showed that HEK293 expresses low levels of mRNAs for ORL1 (cycle threshold, C_t_, 29.3), but little or no mRNAs for MOR (C_t_, undetected), DOR (C_t_, 35.3), and KOR (C_t_, 33.1). As controls, the C_t_ values for hypoxanthine phosphoribosyltransferase 1 (HPRT1) and glyceraldehyde-3-phosphate dehydrogenase (GADPH) in HEK293 were found to be 19.6 and 16.1, respectively. Our quantitative real time PCR results also showed that SH-SY5Y expresses mRNAs for MOR (C_t_, 23.2) and ORL1 (C_t_, 25.8), low levels of mRNAs for DOR (C_t_, 30.9), and no detectable mRNAs for KOR (C_t_, undetected). The C_t_ values for controls were 21.3 for HPRT1, and 15.8 for GADPH. This is consistent with previous studies showing that SH-SY5Y expresses both MOR and DOR proteins at a ratio of approximately 4.5:1 [20].

### Label-free integrative pharmacology profiling and data visualization

We adopted the newly developed iPOT approach to determine the binding and functional selectivity of a family of ligands against the opioid receptor family. This study begun with the preparation of a library consisting of fifty-five opioid ligands, followed by whole cell DMR profiling under different conditions and similarity analysis of respective DMR signals (Table 1). Ligands with interesting label-free profiles were then identified and selected for quantitative pharmacology assessment.

Interrogating SH-SY5Y cells with the library of opioid ligands identified three types of DMR signals (Figure 1a). Out of the fifty-five ligands tested, twenty ligands including naloxone-HCl were silent in this cell line, leading to a net-zero DMR. Conversely, thirty-one ligands including DAMGO produced a positive DMR signal that consists of an initial positive DMR (P-DMR) event followed by a negative DMR (N-DMR) event. The N-DMR event eventually decayed back to a steady level that is still above the baseline at 1 hr poststimulation. The remaining four ligands, DIPPA, etonitazenyl isothiocyanate, BNTX, and ICI 199441, produced a biphasic DMR response whose late N-DMR event eventually decayed below the baseline.

We adopt similarity analysis to classify ligands based on DMR responses. Given that a DMR is a kinetic response and consists of over 200 dimensions due to its high temporal resolution, it is practically impossible to include all time points of a DMR signal for similarity analysis of all ligands under all conditions. Thus, we first reduced the DMR dimensions to three distinct time points (3, 9, and 30 min post-stimulation) for similarity analysis (Figure 1b). This dimensional reduction is based on the clustering of time domains of the DMR responses from all opioid ligands in each of the five cell lines examined. For SH-SY5Y cells, similarity analysis using the unsupervised Ward hierarchical clustering algorithm and Euclidean distance metrics [15,16] showed that all DMR signals with an amplitude greater than 30 pm generally propagate with three distinct time periods: immediate (1–8 min), early (9–15 min), and late responses (15–50 min post-stimulation) (Figure 1c). Clustering based on the entire kinetic response or the reduced three time-points led to similar clusters of ligands in SH-SY5Y cells (comparing Figure 1c with b), although clustering based on the entire kinetic response expectedly gave rise to better resolution than by using the reduced time-points. Therefore, we chose to limit our analysis to the three time point parameters (3, 9, and 30 min) for each DMR response.

### Selectivity of opioid agonists at the opioid receptor family

We first determined the selective agonist activity of opioid ligands in five distinct cell lines using DMR agonist assays, based on their ability to trigger DMR signals in respective cell lines. For the four opioid receptor-expressing cell lines, we included both positive and negative controls to define the range of responses for classification of ligand agonism. For the negative controls (that is, the assay buffer containing equal amount of DMSO), the DMR responses at 9 min poststimulation were found to be 3±12 pm, -4±14 pm, 5±11 pm and 3±5 pm (n = 16) for HEK-MOR, HEK-DOR, HEK-KOR and SH-SH5Y cells, respectively. For the positive controls, the DMR responses at 9 min poststimulation were found to be 240±17 pm, 321±26 pm, 213±21 pm, and 87±9 pm (n = 32) for 10 μM DAMGO in HEK-MOR, 10 μM DPDPE in HEK-DOR, 10 μM BRL52537 in HEK-KOR, and 10 μM DAMGO in SH-SH5Y cells, respectively. For a given cell line, a ligand whose DMR amplitude was within the mean±2σ of its positive control was considered to be a full agonist, while a ligand whose DMR amplitude was smaller the mean-2σ of its positive control and greater than 50 pm was considered to be a partial agonist, and a ligand whose DMR amplitude was smaller than 50 pm was considered to be inactive. A ligand that led to a detectable DMR in HEK293 was viewed to have off-target effect(s).

Table 2 summarizes the agonist activity of all opioid ligands in the five different cell lines. Out of the fifty-five ligands tested, six off-target ligands including BNTX, β-funaltrexamine, etonitazenyl isothiocyanate, ICI 199441, dynorphin A 2–13 and nocicepin 1–13 gave rise to a noticeable DMR in the parental HEK293 cells (Additional file 1: Figure S1). Among the six ligands only BNTX led to an N-DMR in all five cell lines, while the others produced a P-DMR signal in the four opioid receptor-expressing cell lines.

**Table 2:** Classification of opioid ligands based on their DMR agonist activity in the five distinct cell lines

**Name**	**HEK**	**MOR**	**DOR**	**KOR**	**SH-SY5Y**	**Literature classification**
β-Funaltrexamine	off-target	Partial agonist	Partial agonist	Partial agonist		Mu antagonist
BNTX	off-target	off-target	off-target	off-target	off-target	Delta antagonist
Dynorphin A (2–13)	off-target		Partial agonist	Partial agonist		Kappa agonist
Etonitazenyl	off-target	Partial agonist		Partial agonist		Mu agonist
ICI 199,441	off-target	Full agonist	Partial agonist	Full agonist		Kappa agonist
Nociceptin (1–13)	off-target	Partial agonist		Partial agonist		ORL1 agonist
α-Neoendorphin		Partial agonist	Full agonist	Full agonist	Full agonist	Kappa agonist
BRL-52537			Partial agonist	Full agonist		Kappa agonist
BUBUC		Partial agonist	Full agonist	Partial agonist	Partial agonist	Delta agonist
DADLE		Partial agonist	Full agonist	Partial agonist	Full agonist	Delta agonist
DALDA		Partial agonist			Full agonist	Mu agonist
DAMGO		Full agonist	Partial agonist	Partial agonist	Full agonist	Mu agonist
DAMME		Full agonist	Full agonist	Partial agonist	Full agonist	Mu/Delta agonist
Deltorphin II		Partial agonist	Full agonist			Delta agonist
DIPPA		Partial agonist	Partial agonist	Full agonist		Kappa antagonist
DPDPE		Partial agonist	Full agonist	Partial agonist	Full agonist	Delta agonist
DSLET		Full agonist	Full agonist	Partial agonist	Full agonist	Delta agonist
DTLET		Full agonist	Full agonist	Partial agonist	Full agonist	Delta agonist
Dynorphin A (1–13)		Full agonist	Full agonist	Full agonist	Partial agonist	Kappa agonist
Dynorphin A (1–8)		Full agonist	Full agonist	Full agonist	Full agonist	Kappa agonist
Dynorphin B		Partial agonist	Full agonist	Full agonist	Full agonist	Kappa agonist
Endomorphin-1		Partial agonist	Partial agonist		Partial agonist	Mu agonist
Endomorphin-2		Partial agonist	Partial agonist	Partial agonist	Full agonist	Mu agonist
(Leu5)-Enkephalin		Full agonist	Full agonist	Partial agonist	Full agonist	Mu/Delta agonist
(Met5)-Enkephalin		Full agonist	Full agonist	Partial agonist	Full agonist	Mu/Delta agonist
GR 89696		Partial agonist	Partial agonist	Full agonist	Full agonist	Kappa agonist
ICI 204,448		Partial agonist	Partial agonist	Full agonist	Full agonist	Kappa agonist
Levallorphan			Partial agonist	Partial agonist		Partial Mu/delta agonist
Morphiceptin		Partial agonist			Partial agonist	Mu agonist
Nalbuphine		Partial agonist	Partial agonist	Partial agonist		Partial Mu/kappa agonist
Naloxonazine				Partial agonist		Mu antagonist
Naloxone HCl						Opioid antagonist
Naloxone methiodide						Opioid antagonist
Naltrexone				Partial agonist		Opioid antagonist
Naltriben						Delta antagonist
Naltrindole						Delta antagonist
N-Benzylnaltrindole			Partial agonist	Partial agonist		Delta antagonist
N-MPPP		Partial agonist	Partial agonist	Full agonist		Kappa agonist
NNC 63-0532		Partial agonist	Partial agonist	Partial agonist	Full agonist	ORL1 agonist
Nociceptin				Partial agonist		ORL1 agonist
Nor-Binaltorphimine						Kappa antagonist
(−)-Norcodeine		Partial agonist		Partial agonist	Partial agonist	Opioid antagonist
Salvinorin A		Partial agonist		Full agonist		Kappa agonist
SKF10047			Partial agonist	Partial agonist		Opioid agonist/antagonist
SNC 121			Partial agonist	Partial agonist	Partial agonist	Delta agonist
SNC 80		Partial agonist	Partial agonist	Partial agonist	Partial agonist	Delta agonist
Syndyphalin SD-25		Full agonist	Partial agonist	Partial agonist	Partial agonist	Mu agonist
TAPP		Partial agonist	Partial agonist	Partial agonist	Full agonist	Mu agonist
Tramadol		Partial agonist				Weak Mu agonist
(−)-U-50488		Partial agonist	Partial agonist	Full agonist		Kappa partial agonist
(+)-U-50488				Full agonist		Kappa partial agonist
U-50,488H		Partial agonist	Partial agonist	Full agonist		Kappa agonist
U-54494A		Partial agonist	Partial agonist	Partial agonist		Kappa agonist
U-62066		Partial agonist		Full agonist		Kappa agonist
U-69593		Partial agonist	Partial agonist	Full agonist		Kappa agonist

The blank indicates that the ligand did not result in any noticeable DMR.

Out of the fifty-five ligands tested, four ligands including naloxone was inactive in all cell lines, while the other forty-nine ligands gave rise to agonist activity in at least one of the four opioid receptor-expressing cell lines. Several ligands that are believed to be opioid antagonists also produced noticeable DMR in at least one of the engineered cell lines, but not in SH-SH5Y cells. Specifically, nalbuphine and β-funaltrexamine acted as partial agonists at MOR, DOR, and KOR sites, while levallorphan, SKF10047 and N-benzylnaltrindole specific to both DOR and KOR sites, and naloxonazine and naltrexone specific to the KOR.

**Figure 2:**
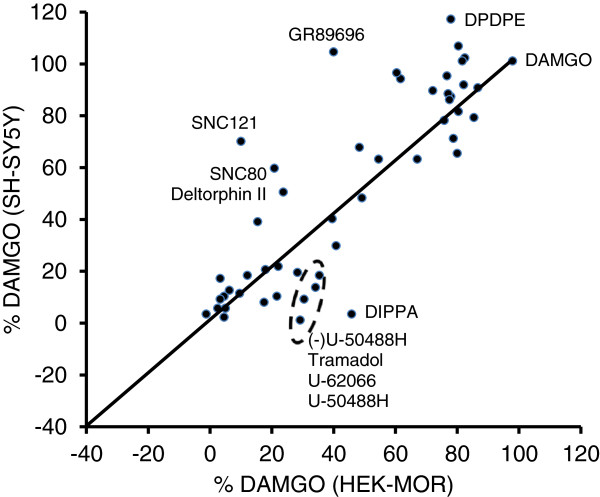
**The correlation analysis of the DMR of forty-nine opiate ligands in HEK-MOR cells versus SH-SY5Y cells after normalized to the DAMGO DMR.** All ligands were assayed at 10 μM.

The pattern of agonist activity in SH-SY5Y cells (Additional file 1: Figure S2) cannot be explained by the solo activation of endogenous MOR, and/or by the differential expression levels of the MOR between SH-SY5Y and HEK-MOR cells. This is expected given that SH-SY5Y expresses both MOR and DOR. This conclusion was supported by correlation analysis between the two cell lines (Figure 2). This analysis excluded the six off-target ligands, and all other responses were normalized to the DAMGO response in respective cell line. Results showed that SNC 121, SNC80 and deltrophin II had no or little activity in the HEK-MOR, but active in SH-SY5Y cells. In contrast, tramadol was active in HEK-MOR, but inactive in SH-SY5Y cells. Similarly, a group of ligands including U-50488H, U62066, DIPPA and (−)U-50488H were active in the three transfected cell lines, but not in SH-SY5Y cells. Furthermore, DPDPE and GR89696 behaved as partial agonists in HEK-MOR cells, but full agonists in SH-SY5Y cells.

### Selectivity of opioid ligands to block the DMR response produced by the activation of opioid receptors

We used a two-step DMR assay (i.e., an antagonist assay) to determine the ability of opioid ligands to block or desensitize the DMR responses resulting from the activation of opioid receptors. The antagonist or desensitization assay was performed in two sequential steps, each lasting about one hour. Cells were pretreated with a ligand from the opioid library, followed by treatment with a fixed dose of a known opioid agonist. A ligand that does not trigger a DMR but blocks the DMR of the known agonist is termed an antagonist. Conversely, a ligand that leads to noticeable DMR response but desensitizes the cells responding to the succeeding agonist is termed an agonist.

**Figure 3:**
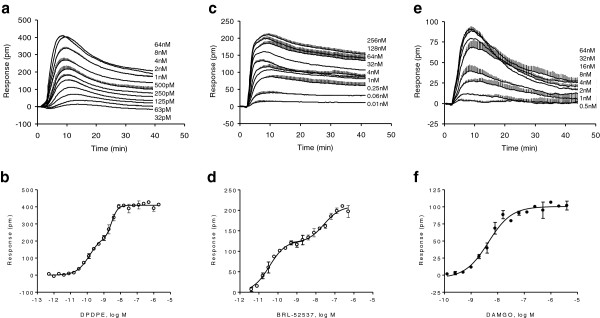
**Dose-dependent responses of agonists in distinct cell lines.** (**a**, **b**) Dose responses of DPDPE in HEK-DOR; (**c**, **d**) Dose responses of BRL-52537 in HEK-KOR; and (**e**, **f**) Dose responses of DAMGO in SH-SY5Y cells. (**a**, **c**, **e**) Real time DMR, each curve represents the mean ± s.d. of 2 independent measurements, each in duplicate (n = 4). (**b**, **d**, **f**) The maximal amplitudes as a function of agonist doses (n = 4).

We first determined the DMR potency of a known agonist for each cell line: DAMGO for HEK-MOR, DPDPE for HEK-DOR, BRL-52537 for HEK-KOR, and DAMGO for SH-SY5Y cells, based on their respective maximal amplitudes. We have previously shown that DAMGO produces a mono-phasic dose response in HEK-MOR cells with an EC_50_ of 0.93±0.12 nM [16]. In HEK-DOR cells, DPDPE produced biphasic dose response with two distinct EC_50_’s of 0.15±0.03 nM, and 2.8±0.09 nM (2 independent measurements, n =4) (Figure 3a and b). In HEK-KOR cells, BRL-52537 also produced a biphasic dose response with two distinct EC_50_’s of 35.6±3.1 pM, and 26.0±1.9 nM (2 independent measurements, n =4) (Figure 3c and d). Conversely, in SH-SY5Y cells DAMGO produced a monophasic dose response with an EC_50_ of 4.5±0.3 nM (2 independent measurements, n =4) (Figure 3e and f).

We next performed cluster analysis of the known agonist DMR responses after pretreatment with the library ligands using unsupervised Ward hierarchical clustering algorithm and Euclidean distance metrics. To achieve high resolution to differentiate the relative potency of opioid ligands to block or desensitize the agonist DMR response at each receptor site we employed a high dose for each agonist tested (10 μM DAMGO for HEK-MOR cells, 10 μM DPDPE for HEK-DOR cells, 10 μM BRL-52537 for HEK-KOR cells, and 10 μM DAMGO for SH-SY5Y cells). The DMR of each known agonist in its respective cell line was shown to be specific to the activation of its respective receptor. Results showed that the cluster analysis separated these ligands into different clusters (Additional file 1: Figure S3), and most of the ligands in each subcluster exhibited DMR characteristics in general agreement with their previously described pharmacology and classifications (Table 2 and Additional file 1: Tables S1–S3).

**Figure 4:**
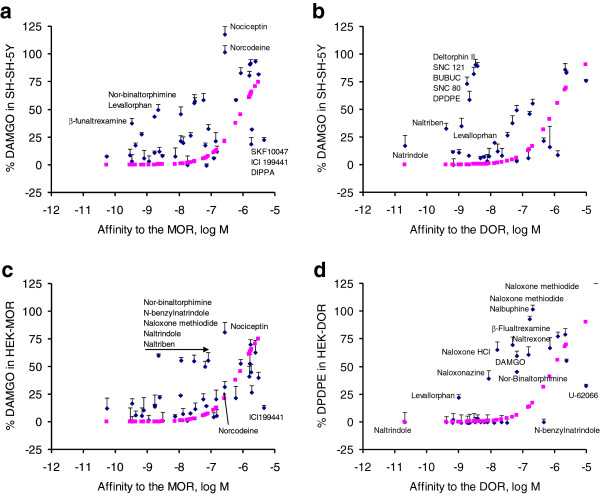
**The inhibition pattern by opioid ligands.** (**a**) The percentage of DAMGO responses in SH-SY5Y cells as a function of the binding affinity of opioid ligands to the MOR. (**b**) The percentage of DAMGO responses in SH-SY5Y cells as a function of the binding affinity of opioid ligands to the DOR. (**c**) The percentage of DAMGO responses in HEK-MOR cells as a function of the binding affinity of opioid ligands to the MOR. (**d**) The percentage of DPDPE responses in HEK-DOR cells as a function of the binding affinity of opioid ligands to the DOR. The percentage of agonist responses after pretreatment with ligands in the library were calculated based on the normalization of the agonist response in the presence of a ligand to the positive control (*i.e.*, the agonist response after pretreatment with the vehicle buffer only). The data points in pink were calculated based on the known binding affinity of each ligand against the specific receptor using% agonist response = 1/[1 + 10^log (X – K_i_)], wherein X is the concentration of each ligand, and K_i_ the binding affinity obtained in literature. Included in this analysis are ligands whose binding affinities at specific receptor sites are known.

We further examined the DMR responses of DAMGO in SH-SY5Y cells with and without pretreatment with the library ligands, based on reported affinities of opioid ligands (Additional file 1: Tables S1 to S3) [39]. Results show that the ligands blocking the DAMGO-elicited DMR in HEK-MOR also blocked the DAMGO DMR in SH-SY5Y cells, suggesting that the DAMGO response in SH-SY5Y is mostly originated from the activation of the MOR. However, the extent of the DAMGO-induced DMR observed after pretreatment with the library of opioid ligands in SH-SY5Y cells cannot be explained by the known affinities of these ligands binding to MOR or the DOR sites (Figure 4a and b). To best illustrate this, we first assumed that the DAMGO DMR in SH-SY5Y cells is originated from the activation of MOR or DOR alone, and then compared the actual DAMGO response with the calculated one for each ligand based on its reported affinity for the MOR (Figure 4a) or DOR (Figure 4b), respectively. This analysis showed that three potent MOR antagonists, β-funaltrexamine, levallorphan and nor-binaltorphimine, appeared to be less potent to block the DAMGO-induced DMR in SH-SY5Y cells than that would be expected at MOR binding sites; conversely, three agonists including SKF10047, ICI 199,441 and DIPPA desensitized SH-SY5Y cells with greater potency than their reported affinities at the MOR, and the remaining ligands gave rise to expected results (Figure 4a). This suggests that the DAMGO response has additional signaling component beside the MOR. Further, the DOR-selective agonists including deltorphin II, SNC121, BUBUC, SNC80 and DPDPE desensitized SH-SY5Y cells with lower potency than that would be expected at DOR binding sites, but the rest ligands behaved as expected at DOR binding sites (Figure 4b), suggesting that the DAMGO response in SH-SY5Y cells has additional signaling component beside the DOR. As comparison, the DAMGO induced DMR in HEK-MOR cells after pretreatment with library ligands was correlated well with their known binding affinities, with an exception of a group of antagonists including nor-binaltorphimine, N-benzylnatrindole, naloxone methiodide, naltrindole, and naltriben (Figure 4c). Similarly, the DPDPE-induced DMR in HEK-DOR cells after the ligand pretreatment was mostly correlated well with their known binding affinities, except for a group of opioid antagonists including naloxone HCl (Figure 4d). The partial blockage of the DAMGO response in HEK-MOR, or of the DPDPE response in HEK-DOR by these antagonists is partially due to the use of high dose agonists used (10 μM for both agonists). Other factors such as receptor dimerization or differing cellular contexts may also contribute to these differences. Nonetheless, these results suggest that ligand pharmacology at the whole cell level is different from the *in vitro* binding profiles.

### Functional selectivity of opioid ligands at the opioid receptors

We hypothesized that functional selectivity of a ligand at the whole cell level is reflected by the sensitivity of its DMR response to pretreatment of cells with various probe molecules [15,16]. We excluded BNTX, β-funaltrexamine, etonitazenyl isothiocyanate, ICI 199441, dynorphin A2-13 and nocicepin 1–13 from biased agonism analysis because of their off-target activity. To effectively visualize the effect of the probe pretreatments, we used the net change of the DMR response of a ligand (*i.e.*, Its DMR in a probe molecule pretreated cells minus its DMR in DMSO treated cells) for similarity analysis. This was done for all assay conditions except for PTX pretreatment wherein the raw DMR were used, since these DMR are generally small with amplitudes similar to the net change in other probe-treated cells – an important consideration for accurate clustering. The DMR in the DMSO treated cells were also included as references. A positive net change indicates that the probe pretreatment potentiates a ligand-induced DMR response, while a negative net change indicates a decrease in a ligand-induced DMR response by the probe pretreatment. The averaged responses of at least 2 experiments were used. Statistical analysis showed that for a total of 2× 3960 DMR data points obtained (3 cell lines × 8 assay conditions × 55 ligands × 3 time points), 97.1% gave rise to an absolute difference between replicates for a ligand under one condition that was smaller than 10 pm, and the remaining 2.9% (115 parameters, all of which occurred in either HEK-DOR or HEK-KOR cells) was between 10 and 20 pm. Thus, a net change induced by a probe pretreatment greater than 30 pm was considered to be significant for both HEK-DOR and HEK-KOR cells, while a net change greater than 20 pm was to be significant for SH-SY5Y cells.

**Figure 5:**
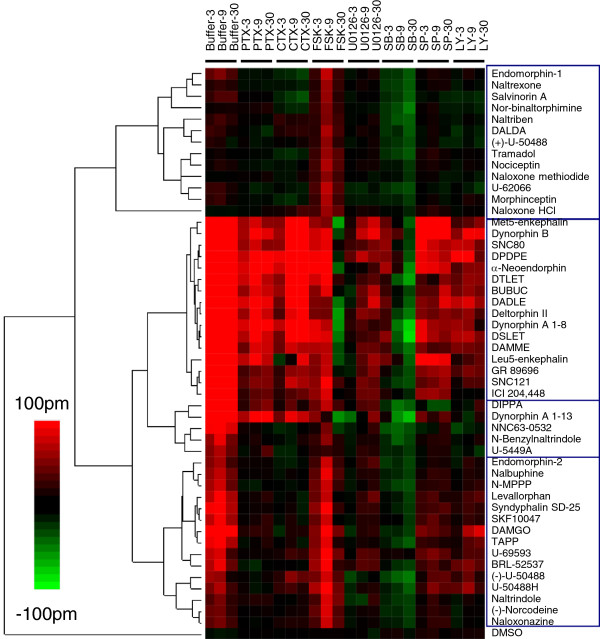
**A colored heat map based on the functional selectivity of opioid ligands at the DOR.** The DMR signals of ligands in HEK-DOR cells with and without (*i.e.*, buffer) pretreatment with probe molecules including PTX, CTX, U0126, SB202190, SP600125 and LY294002 were used to generate the heat map. All of the ligands in the library were assayed at 10 μM. The negative control (DMSO) was also included. To effectively visualize the impact of probe molecules, the net change for each ligand after pretreatment was obtained via subtraction, except for both the positive control (*i.e.*, DMR in cells pretreated with the buffer vehicle only) and the ligand DMR in PTX-pretreated cells for which the raw data were used.

Profiling HEK-DOR cells after pretreatment with seven probe molecules produced a heat map which grouped the ligands into two large superclusters (Figure 5). Notably, all ligands gave rise to a P-DMR response under at least one assay condition. The first supercluster consists of antagonists and ligands that were inactive in the untreated HEK-DOR cells, except for endomorphin-1 which acted as a partial agonist in the control HEK-DOR cells (*i.e.*, the cells pretreated with the vehicle only). All ligands in this supercluster exhibited a small P-DMR in the forskolin-pretreated cells, suggesting that these ligands gave rise to weak partial agonist activity when the basal cAMP level is high. The second supercluster can be further subdivided into three subclusters, one for ligands such as DPDPE who appear to act as full agonists, and two others comprised of ligands that appear to act as partial agonists. For the full agonist subcluster, these ligands still triggered a noticeable DMR response in PTX-pretreated cells; CTX pretreatment generally increased their DMR; forskolin only increased their early DMR response but suppressed their late DMR response (*i.e.*, 30 min post-stimulation); U0126, SP600125 and LY294002 generally increased their DMR; but SB202190 suppressed their DMR. The second subcluster was comprised of DIPPA, dynorphin A 1–13, NNC63-0532, N-benzylnaltrindole, and U-5449A, all of which were insensitive to U0126, SP600125 and LY294002 pretreatment. However, only DIPPA and dynorphin A 1–13 produced a noticeable DMR response in PTX treated cells and led to an increased DMR in the CTX or forskolin treated cells. Forskolin pretreatment selectively suppressed the late DMR of dynorphin A 1–13, and SB202190 only suppressed the DMR of DIPPA, dynorphin A 1–13, NNC63-0532, N-benzylnaltrindole. The third subcluster consists of fifteen ligands including endomorphin-2, none of which produced any DMR response in PTX- treated cells. All ligands in this subcluster were insensitive to the pretreatment with CTX, U0126 or SB202190, but were increased by forskolin pretreatment. Together, these results suggest that the opioid ligands are divergent in their biased agonism at the DOR.

**Figure 6:**
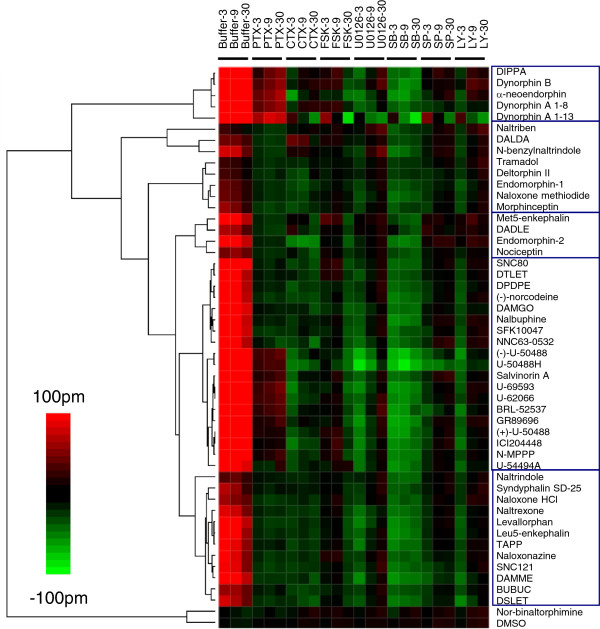
**A colored heat map based on the functional selectivity of opioid ligands at the KOR.** All of the ligands in the library were assayed at 10 μM. The negative control (DMSO) was also included. To effectively visualize the impact of probe molecules, the net change for each ligand after pretreatment was obtained via subtraction, except for both the positive control (*i.e.*, DMR in cells pretreated with the buffer vehicle only) and the ligand DMR in PTX-pretreated cells for which the raw data were used.

The DMR profiles obtained in HEK-KOR cells under the eight assay conditions produced a heat map that also separated the ligands into two superclusters (Figure 6). The first cluster consists of the DMSO negative control and nor-binaltorphimine. The absence of any DMR under all conditions suggests that nor-binaltorphimine behaved as a true neutral antagonist at the KOR. The second supercluster can be further subdivided into multiple subclusters, each of which produced a P-DMR signal under at least one assay condition. Agonists that produced a detectable P-DMR in the PTX pretreated cells include DIPPA, dynorphin B, α-neoendorphin, dynorphin A 1–8, dynorphin A 1–13, (−)-U-50488, U-50488H, salvinorin A, U-69595, U-62066, BRL-52537, and GR89696. Unlike the situation in HEK-MOR and HEK-DOR cells, the DMR responses of almost all agonists were found to be insensitive to both CTX- and forkolin-pretreatment in HEK-KOR cells. A similar pattern was observed for both SP600125- and LY294002-treatment. However, pretreatment of HEK-KOR cells with SB202190 suppressed the ligand-library DMR response induced by virtually all agonists, with U-50488H exhibiting the most significant suppression. Further, U0126 selectively suppressed the DMR of (−)-U-50488 and U-50488H. Together, these results suggest that p38 MAPK pathway may play a more significant role in the KOR signaling than any of the other kinase pathways.

**Figure 7:**
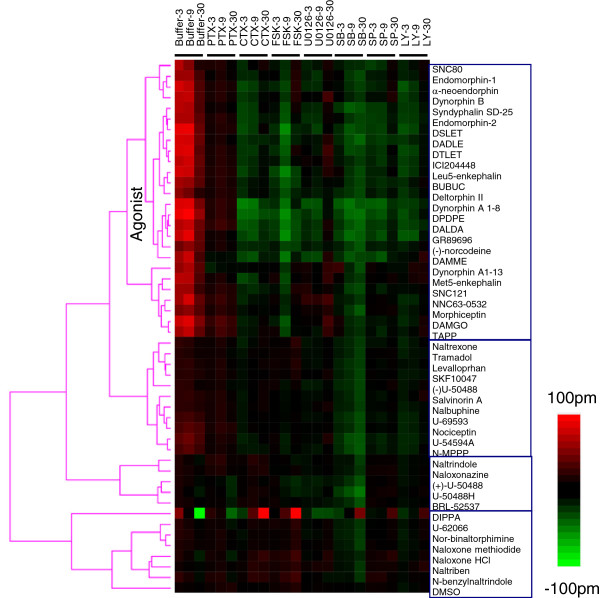
**A colored heat map based on the functional selectivity of opioid ligands at the endogenous receptor in SH-SY5Y cells.** All of the ligands in the library were assayed at 10 μM. The negative control (DMSO) was also included. To effectively visualize the impact of probe molecules, the net change for each ligand after pretreatment was obtained via subtraction, except for both the positive control (*i.e.*, DMR in cells pretreated with the buffer vehicle only) and the ligand DMR in PTX-pretreated cells for which the raw data were used.

We next profiled the opioid library ligands using SH-SY5Y cells under the eight different assay conditions. Results showed that SH-SY5Y cells led to different patterns for the library ligands (Figure 7). Ligands in the agonist supercluster typically behaved as would be expected. However, some ligands, most notably DIPPA, produced unique DMR responses – DIPPA triggered a biphasic DMR response which eventually decayed below the baseline in the native SH-SY5Y cells, while PTX pretreatment suppressed both the early and late DMR response; both CTX and forskolin potentiated the DMR response; U0126 converted the DMR response to a single phase N-DMR; and SB202190 delayed the time to reach its peak. This unique pattern suggests that DIPPA activates both G_αi_-dependent and independent pathways. Except for DAMGO and TAPP, ligands in the agonist supercluster led to little or no DMR in the PTX-treated cells. Both CTX and forskolin suppressed the DMR of dynorphin A 1–8, DPDPE or DALDA. Forskolin also suppressed the DMR of Leu5-enkephalin, DSLET and DAMME. In general, the kinase inhibitors mostly suppressed the same group of agonists which included dynorphin A 1–8, DPDPE, DALDA, GR89696 and DAMME. These results suggest that ligand pharmacology in SH-SY5Y cells is distinct from those in both HEK-MOR and HEK-DOR.

### Potency and efficacy of opioid ligands at distinct opioid receptors

**Figure 8:**
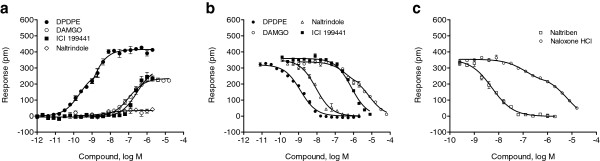
**Dose responses of a panel of opioid ligands in HEK-DOR cells.** (**a**) Dose dependent responses of opioid ligands obtained using DMR agonist assays. The maximal amplitudes were plotted as a function of agonist doses. (**b**) Dose-dependent desensitization by the DOR agonists of HEK-DOR cells to the repeated stimulation with DPDPE at 64 nM. (**c**) Dose-dependent inhibition by the DOR antagonists of HEK-DOR cells to the succeeding stimulation with DPDPE at 64 nM. For (**b**) and (**c**) the maximal amplitudes of the DPDPE DMR were plotted as a function of ligand doses, and data represents the mean ± s.d. for 2 independent measurements, each in duplicate (n = 4).

Based on the iPOT profiles, we further examined the dose responses of selected ligands at distinct opioid receptors. For HEK-DOR cells, besides DPDPE five additional ligands were profiled using both DMR 1-step agonist and 2-step antagonist assays. The agonist DMR assays showed that four of these ligands including DPDPE, DAMGO, ICI 199441 and naltrindole, gave rise to dose-dependent responses in HEK-DOR cells (Figure 8a) while naltriben and naloxone HCl were silent in HEK-DOR cells. DPDPE resulted in a biphasic dose response, resulting to two saturable amplitudes, 279±11 pm and 415±17 pm (n =16), respectively (Figure 3a and b). However, all other agonists led to a monophasic dose response, yielding an EC_50_ of 281.1±21.3 nM ( n = 4), 104.8±4.9 nM (n =4) and 6.1±0.9 nM (n =4) for ICI 199441, DAMGO and naltrindole, respectively (Figure 8a). The corresponding maximal amplitudes were found to be 300±23 pm (n =16), 235±13 pm (n =16) and 82±9 pm (n =16).

The DMR antagonist assay showed that distinct ligands differentially blocked the succeeding DPDPE-induced DMR response in HEK-DOR cells (Figure 8b and c). The dose-dependent desensitization by DPDPE is best fitted with single phase sigmoidal non-linear regression, leading to an IC_50_ of 1.25±0.10 nM (n = 4). Similar monophasic inhibitory dose-responses were obtained for naltrindole (IC_50_, 8.87±0.39 nM; n = 4), ICI 199441 (IC_50_, 753±67 nM; n = 4), and naltriben (IC_50_, 5.30±0.36 nM; n = 4). However, a biphasic dose-dependent inhibition of the DPDPE-induced DMR was observed for the partial agonist DAMGO (IC_50_: 368±51 nM and 7.58±1.32 μM; n =4), and the antagonist naloxone HCl (IC_50_: 135.1±14.9 nM and 6.41±0.75 μM; n =4).

**Figure 9:**
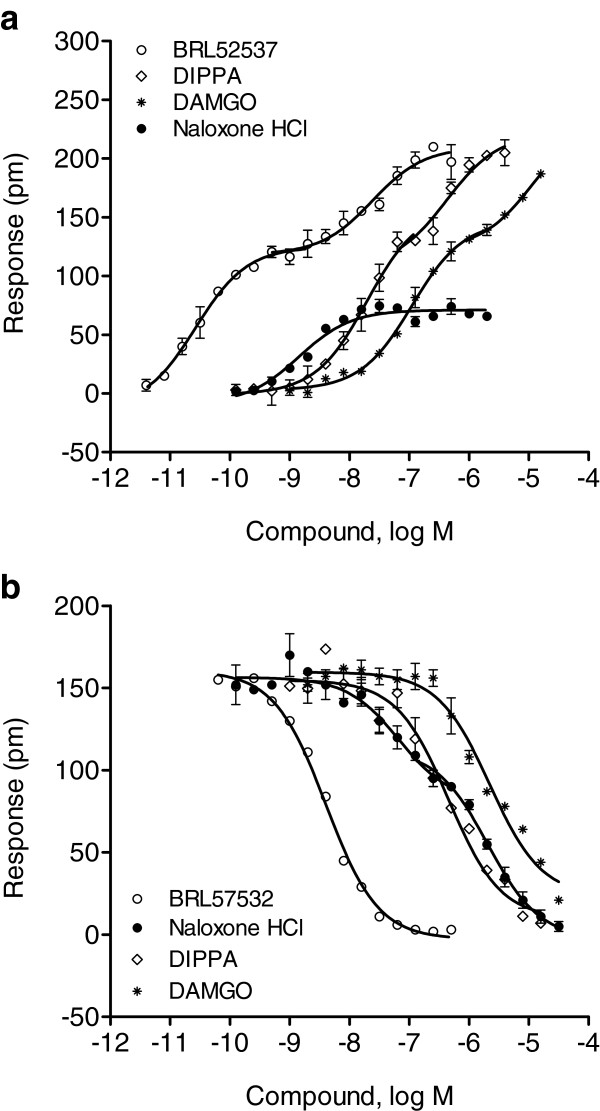
**Dose responses of a panel of opioid ligands in HEK-KOR cells.** (**a**) Dose dependent responses of opioid ligands obtained using DMR agonist assays. The maximal amplitudes were plotted as a function of agonist doses. Data represents the mean ± s.d. for 2 independent measurements, each in duplicate (n = 4). (**b**) Dose-dependent inhibition of the DMR of 64 nM BRL-57532 by opioid ligands. The maximal amplitudes of the BRL-57532 DMR were plotted as a function of ligand doses. Data represents the mean ± s.d. for 2 independent measurements, each in duplicate (n = 4).

We next characterized the KOR using four opioid ligands, including BRL-52537. The DMR agonist assay showed that all four ligands triggered dose-dependent DMR signals (Figure 9a), similar to BRL-52537 (Figure 3b). Like BRL-52537, DIPPA and DAMGO all yielded biphasic dose responses. This analysis revealed EC_50_ values of 13.4±1.5 nM and 239.6±11.2 nM (n = 4) for DIPPA, and 93.8±7.4 nM and 4.5±1.1 μM (n =4) for DAMGO. The two saturable amplitudes were 120±6 pm and 207±13 pm (n = 4) for BRL-52537, 138±8 pm and 205±9 pm (n = 4) for DIPPA, and 139±6 pm and 200±8 pm (n = 4) for DAMGO. In contrast, the partial agonist naloxone HCl yielded a monophasic dose response with an EC_50_ of 1.4±0.2 nM, with a maximal response of 69±5 pm (n = 4). Further, the two-step DMR antagonist assay showed that distinct ligands differentially inhibited the HEK-KOR cells responding to repeated stimulation with 64 nM BRL-52537 (Figure 9b). DIPPA, DAMGO and BRL-52537 each inhibited the BRL-52537 response with single phase sigmoidal non-linear regression producing IC_50_ values of 454.9±32.3 nM (n = 4), 2.21±0.51 μM, and 4.1±0.23 nM, respectively. In contrast, the dose-dependent inhibition by the antagonist naloxone HCl was best fitted with a biphasic sigmoidal non-linear regression, which exhibited biphasic IC_50_’s of 67.2±5.6 nM and 2.05±0.0.54 μM (n = 4).

**Figure 10:**
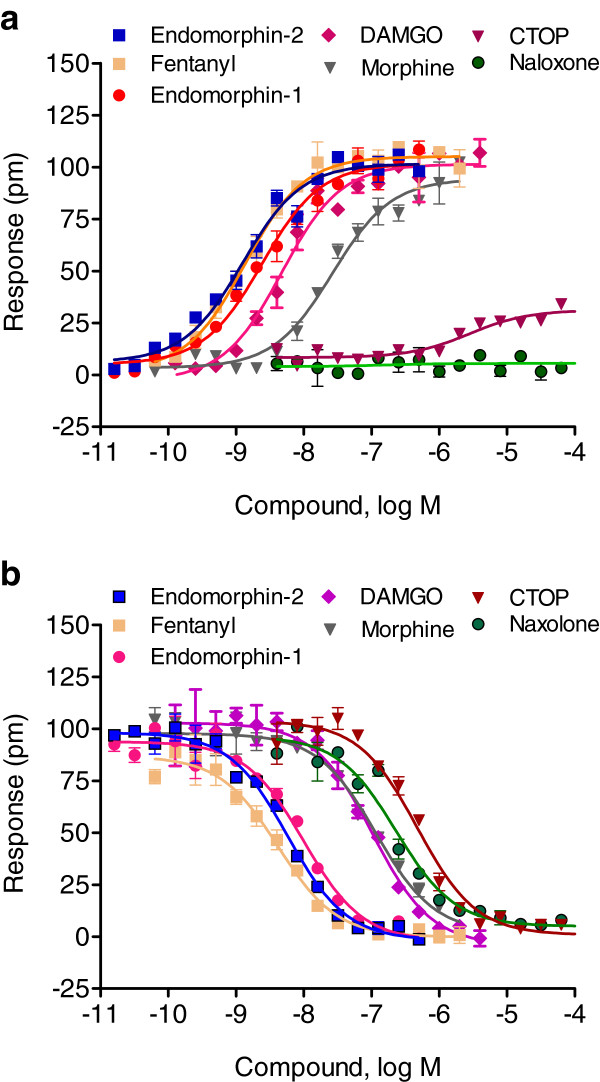
**Dose responses of a panel of opioid ligands in SH-SY5Y cells.** (**a**) Dose dependent responses of opioid ligands obtained using DMR agonist assays. The maximal amplitudes were plotted as a function of agonist doses. (**b**) Dose-dependent inhibition of the DMR of 64 nM DAMGO by opioid ligands. The maximal amplitudes of the DAMGO DMR were plotted as a function of ligand doses. For (**a**) and (**b**) data represents the mean ± s.d. for 2 independent measurements, each in duplicate (n = 4).

Lastly, we characterized the DMR response elicited by opioid receptors in SH-SY5Y cells utilizing seven known agonists and antagonists. Results from the DMR agonist assay showed that all ligands yielded dose-dependent P-DMR signals, except for naloxone HCl, which did not produce any observable DMR response in SH-SY5Y cells (Figure 10a). Similar to DAMGO (Figure 3e), the dose-dependent activation responses were best fitted using a single phase sigmoidal non-linear regression, revealing EC_50_ values of 26.5±2.1 nM (n = 4), 1.4±0.2 nM (n = 4), 2.4±0.2 nM (n = 4), 1.2±0.1 nM (n = 4), and 2.8±0.3 nM (n = 4) for morphine, fentanyl, endomorphin-1, endomorphin-2 and CTOP, respectively (Figure 10a). The maximal DMR responses were found to be 102±8 pm, 94±5 pm, 105±7 pm, 102±6 pm, 102±7 pm, and 31±4 pm (n = 16 for all) for DAMGO, morphine, fentanyl, endomorphin-1, endomorphin-2 and CTOP, respectively. The two-step DMR antagonist assay showed that all ligands blocked the DMR produced by 64 nM DAMGO in a dose-dependent fashion. Single IC_50_ values of 1.0±0.1 nM, 115.8±14.7 nM, 4.2±0.3 nM, 10.0±0.9 nM, 5.8±0.4 nM, 475.5±39.7 nM, and 231.4±21.5 nM were obtained for DAMGO, morphine, fentanyl, endomorphin-1, endomorphin-2, CTOP, and naloxone HCl, respectively (Figure 10b). Together, these results suggest that the family of opioid receptors exhibit complex pharmacology.

## Discussion

Functional selectivity represents the underlying basis for drug selectivity, one of the most important pharmacological properties of drug molecules which assist to determine their *in vivo* efficacy and therapeutic index. However, functional selectivity has not been fully integrated into the mainstream drug discovery and development processes. This is partly because of the simplistic molecular assays conventionally used to characterize the pharmacological properties of many drug molecules and partly because of unknown molecular mode(s) of action that is critical to *in vivo* efficacy or *in vivo* side effects of drugs. This issue is exemplified by opioid ligands. Molecular assays have revealed a wide array of biased agonism demonstrated by opioid ligands which appear to be cell-systems and assay technology dependent [5,6,34]. However, multidimensional biased agonism makes it difficult to rank candidate compounds for *in vivo* testing and to relate a specific biased agonism of drug molecules to their *in vivo* profiles.

Many opioid ligands often display relatively poor selectivity binding to different opioid receptor family members [39,40]. This problem is exacerbated by the fact that the binding affinity profiles of opioid ligands do not directly translate into their selectivity in cellular and *in vivo* environments due to the expression of more than one opioid receptor or its splice variants in native cells, as well as the possibility that opioid receptors may present in different oligomerizational states [41-45]. Thus, an effective means to differentiate drug candidate molecules based on both binding and functional selectivity in native cells would be beneficial to identify and prioritize lead compounds, and to relate *in vitro* results to *in vivo* profiles.

Recently, we have developed a label-free iPOT approach and applied it to differentiate individual ligands in libraries for both β_2_-adrenergic receptor [15] and the MOR [16]. High resolution heat maps obtained allowed us to sort these ligands into distinct clusters based on their cellular binding profiles and pathway biased agonism. Here, we extended this approach to survey the entire classic opioid receptor family (mu-, kappa- and delta-receptors). Both recombinant and native cells expressing opioid receptors were used to generate DMR profiles of a library of opioid ligands using a battery of DMR assay formats. The DMR profiles for all the ligands were translated into numerical coordinates which were subject to similarity analysis to determine the similarity and distance between ligand pairs. The results obtained were visualized using a color-coded heat map with a distance-dendrogram. Here, a variety of probe molecules such as a kinase inhibitor (e.g., SB202190) were used to pretreat the cells in order to manifest the sensitivity of a ligand-induced DMR to the altered cellular background. Such sensitivity is primarily used as a differentiating factor for ligand classification, rather than for determining the exact cellular mechanism of functional selectivity at a specific pathway (e.g., p38 MAPK activation). This is because kinase inhibitors such as SB202190 are known to inhibit multiple targets, and DMR is a whole cell response. On the other hand, MOR, KOR and DOR all can result in p38 MAPK activation in a cell context dependent manner, and kinase cascades have been proposed to be a basis to differentiate ligand-directed signaling at opioid receptors [34]. Our data indicate that compared to those in HEK-MOR, HEK-DOR and native SH-SH5Y cells, the DMR of almost all agonists in the HEK-KOR cells exhibited much higher sensitivity to the SB202190 pretreatment, suggesting that p38 MAPK pathway may be more important in the KOR signaling. However, the biological implications still need further elucidation.

This methodology led to several interesting findings. First, the off-target activity of a subset of ligands including BNTX, β-funaltrexamine, etonitazenyl isothiocyanate, ICI 199,441, dynorphin A 2–13 and nociceptin 1–13 was visualized in both HEK293 and SH-SY5Y cells, indicating that DMR assays are indeed capable of characterizing molecules with much wider pathway coverage than conventional pharmacological or molecular assays.

Second, opiate ligands were found to display distinct pharmacology in MOR or DOR stably expressed cell lines versus the native SH-SY5Y cells. Such a cellular background-dependent pharmacology, termed phenotypic pharmacology, is common to many GPCR ligands [46], and is believed to be originated from many different factors [47-49]. The specific cellular mechanisms causing the differential pharmacology of these ligands in different opioid receptor expressing cells are unknown, and further studies are warranted.

Third, almost all ligands in the library behaved as agonists in at least one opioid receptor expressing cell line with or without pretreatment with probe molecules. Using the DMR agonist assay, we found that out fifty-five opioid ligands testes, forty-nine displayed agonist activity in at least one opioid receptor-expressing cell line (Table 2). This is significant since as many as thirteen ligands in the library were classified as opioid receptor antagonists (Table 2, Additional file 1: Tables S1–S3). Furthermore, all ligands displayed agonist activity in at least one opioid receptor expressing cell lines under one condition. In HEK-KOR cells, nor-binaltorphimine was distinct as it did not trigger any DMR response under any conditions, leading us to conclude that nor-binaltorphimine was a true neutral antagonist for the KOR.

Fourth, pathway biased agonism was also visualized for many ligands. First, G_αi_-independent signaling was evident in the DMR produced by a subset of ligands in PTX-treated cells. Generally full agonists and strong partial agonists for each receptor led to a detectable DMR response in PTX-treated cells, indicative of activating G_αi_-independent signaling. An alternative mechanism is that PTX treatment unnaturally shifts receptor signaling to a different signal transduction pathway. As such the lack of a complete blockade of signal with PTX does not necessarily mean that under naïve conditions (when functional G_αi_ is present) a ligand signals through G_αi_ -independent pathways. Second, CTX and forskolin pretreatment generally increased the DMR response induced by opioid agonists in both HEK-MOR [16] and HEK-DOR cells (Figure 5), but clearly suppressed the DMR of a subset of opioid ligands in HEK-KOR and SH-SY5Y cells (Figures 6 and 7, respectively). These patterns suggest that the KOR in HEK-KOR cells and the opioid receptors in SH-SY5Y may also signal via a pathway distinct from G_αi_.

Lastly, the iPOT analysis of opioid ligands further indicates the complexity of opioid ligand pharmacology. First, the difference in ligand specificity between HEK-MOR and SH-SY5Y cells, or between HEK-DOR and SH-SY5Y cells (Figure 4) cannot be explained solely by the known affinity of these ligands for the MOR or for the DOR, respectively [39]. Such a difference seems to be reflective of the presence of different populations of endogenous opioid receptors or the different level and complement of second messengers and signal transduction components in SH-SY5Y cells. Second, the dose-dependent efficacy and potency of panels of ligands to activate opioid receptors, together with the dose-dependent desensitization/inhibition of the activation of opioid receptors, clearly shows that different ligands produce very different types of dose responses. These responses may be monophasic or biphasic in a ligand- and cell-dependent manner. The biphasic dose responses in opioid receptor expressing cells observed for certain agonists may be related to dual modes of action of the ligands acting at a receptor; that is, the ligands at low doses are biased to a specific pathway, but at higher doses the ligands activate a broader range of pathways [50]. Alternatively, a biphasic dose response for agonists and antagonists may be associated with the existence of different receptor states such as functional monomers and oligomers [51]. A ligand may have different potency to activate or deactivate distinct receptor populations. Nonetheless, the present study represents the first study using label-free cellular assays to assess the binding and functional selectivity of opioid ligands across the entire classic opioid receptor family.

We are still at the early phase to understand how label-free mirrors the innate complexity of drug-target interactions in living cells or cell systems. To elucidate biased agonism, several different approaches have been proposed. Owing to wide pathway coverage, label free is quickly realized to be able to manifest the biased agonism through producing pathway-dependent variations in the whole cell phenotypic profile of different ligands [15]. Multi-parameter analysis based on kinetics can be used to sort ligands into different clusters [13]. Profiling of the same set of ligands in different cellular backgrounds has been attempted to determine biased agonism [52], while comparing label-free with molecular assay results also manifests biased agonism within the same cell background [53]. Controlling the duration of agonist exposure and receptor resensitization using microfluidics offers additional levers to determine ligand-directed functional selectivity [54]. The iPOT approach represents the next step toward deeper and broader elucidation of the biological complexity of drug-target interactions. This approach leverages the signaling capacity of a receptor and the sensitivity of label-free profiles to cell preconditioning via pathway modulation. Owing to the same measurement (that is, the label-free profiling), similarity analysis can be performed and used to sort ligands into different clusters based on their ontarget and off-target pharmacology. A rationale way for lead selection based on the iPOT is to select a few representative ligands from each cluster for *in vivo* testing. The future of label-free is dependent on the identification of an *in vitro* label-free profile that is linked to the *in vivo* action of drug molecules. Nonetheless, the high resolution heat maps and pharmacological characterization of the opioid receptor family using DMR response assays suggest that the iPOT is powerful new approach for elucidating of the complex and multifaceted efficacy of GPCR ligands, and label-free cellular assays are uniquely sensitive to the complexities of receptor mediated signal transduction at the whole-cell level, and as such inform the process of drug discovery in ways that other assay technologies cannot. The power of the iPOT to differentiate ligands can be further improved by using optimizing the algorithm for similarity analysis, in particular methods that take both time domain and signal amplitude into account [35]. The iPOT approach offers a unique platform for drug development when functional selectivity is important.

## Conclusions

In conclusion, we have applied label-free DMR whole cell profile-centred iPOT approach to systematically survey a fifty-five ligand library against the opioid receptor family members in both native and engineered cell backgrounds. The off-target activity, binding and functional selectivity of these ligands have been clearly evident. Notable is that all ligands display certain agonist activity under specific conditions, and opioid ligands exhibit complex pharmacology in both receptor and cell background dependent manner. These label-free profiling results also suggest the necessity to reclassify the ligands. The profiling approach presented here may be useful for lead compound selection.

## Abbreviations

Ct: Cycle threshold; CTX: Cholera toxin; DMR: Dynamic mass redistribution; DOR: Delta opioid receptor; GADPH: Glyceraldehyde-3-phosphate dehydrogenase; GPCR: G protein-coupled receptor; HPRT1: Hypoxanthine phosphoribosyltransferase 1; iPOT: Integrative pharmacology on-target; KOR: Kappa opioid receptor; MOR: Mu opioid receptor; ORL1: Opioid-like receptor-1; PTX: Pertussis toxin

## Competing interests

ET, and YF are employees and shareholders of Corning Inc. HS was an employee of Corning Inc. MM and RL received sponsored research funding from Corning Inc. Epic system is a marketed product. DMR assays are patented. There are no other patents, products in development, or marketed products to declare. This does not alter the authors’ adherence to all the BMC Pharmacology policies on sharing data and materials.

## Authors’ contributions

YF, MM, RL conceived and designed the experiments; MM, SH, ET performed the experiments; MM and YF analyzed the data; MM, HS, ET, YF, RL contributed reagents/materials/analysis tools; YF, MM, RL wrote the paper; YF designed the software used in analysis. All authors have read and approved the manuscript.

## Pre-publication history

The pre-publication history for this paper can be accessed here:

http://www.biomedcentral.com/2050-6511/14/17/prepub

## Supplementary Material

Additional file 1: Figure S1 DMR characteristics of a subset of ligands in parental HEK293 cells, **Figure S2**: A colored heat map based on the DMR of opioid ligands in five different cell lines, **Figure S3**: A colored heat map based on the selectivity of opioid ligands to block the DMR of control agonists in respective cell lines, **Table S1**: Opioid ligands and their affinity binding to the MOR, **Table S2**: Opioid ligands and their affinity binding to the DOR, and **Table S3**: Opioid ligands and their affinity binding to the KOR. Click here for file
