# Supplementary material for: Label-free integrative pharmacology on-target of opioid ligands at the opioid receptor family
Source: BMC Pharmacol Toxicol. 2013 Mar 12;14:17. doi: 10.1186/2050-6511-14-17 (PMC3602246; doi:10.1186/2050-6511-14-17)
Supplement: Additional file 1: Figure S1 — DMR characteristics of a subset of ligands in parental HEK293 cells, Figure S2: A colored heat map based on the DMR of opioid ligands in five different cell lines, Figure S3: A colored heat map based on the selectivity of opioid ligands to block the DMR of control agonists in respective cell lines, Table S1: Opioid ligands and their affinity binding to the MOR, Table S2: Opioid ligands and their affinity binding to the DOR, and Table S3: Opioid ligands and their affinity binding to the KOR. [file 2050-6511-14-17-S1.pdf]

Supplementary Information

Label-free integrative pharmacology on-target of opioid ligands at the opioid receptor family

Megan Morse[1], Haiyan Sun[2], Elizabeth Tran[2], Robert Levenson[1,*] and Ye Fang[2,*]

[1.] *Department of Pharmacology, Pennsylvania State University College of Medicine, Hershey, Pennsylvania, United States of America*

[2.] *Biochemical Technologies, Science and Technology Division, Corning Inc., Corning, New York, United States of America*

* Robert Levenson, Email: rlevenson@hmc.psu.edu; Ye Fang, Email: fangy2@corning.com

Figure S1 DMR characteristics of a subset of ligands in parental HEK293 cells.

Figure S2 A false colored heat map based on the DMR of opioid ligands in five different cell lines.

Figure S3 A false colored heat map based on the selectivity of opioid ligands to block the DMR of control agonists in respective cell lines.

Table S1 Opioid ligands and their affinity binding to the MOR.

Table S2 Opioid ligands and their affinity binding to the DOR.

Table S3 Opioid ligands and their affinity binding to the KOR.

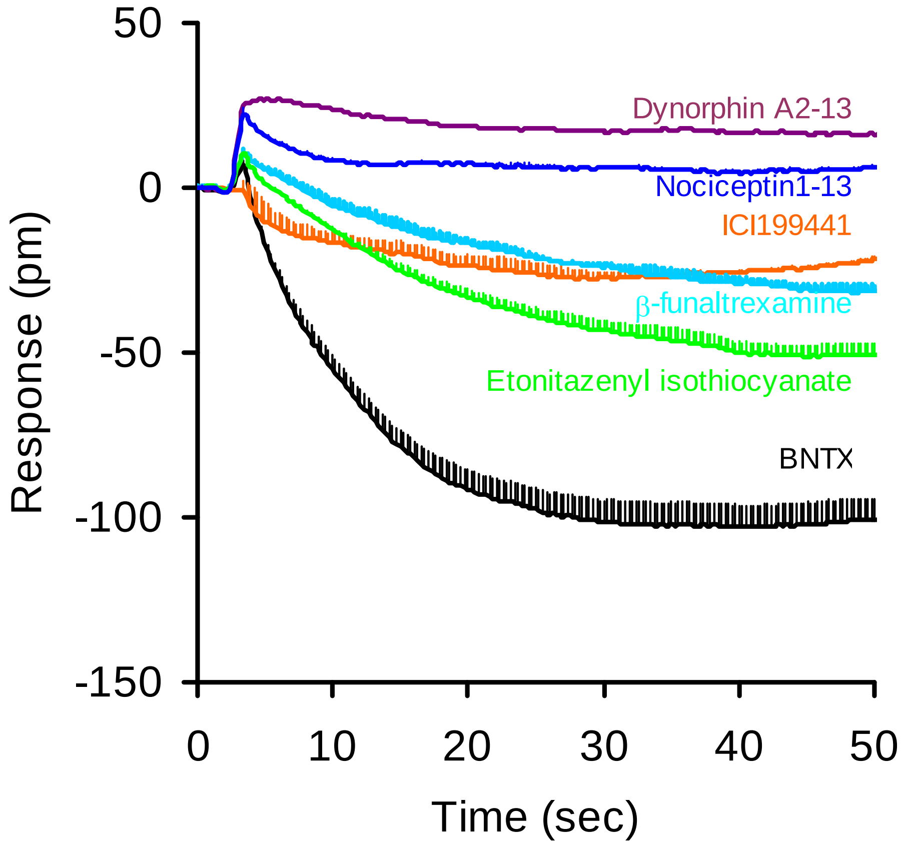

Figure S1. DMR characteristics of a subset of ligands in parental HEK293 cells. The DMR was due to the activation of an unknown endogenous target(s). Data represents the mean ± s.d. for 2 independent measurements (n=4).

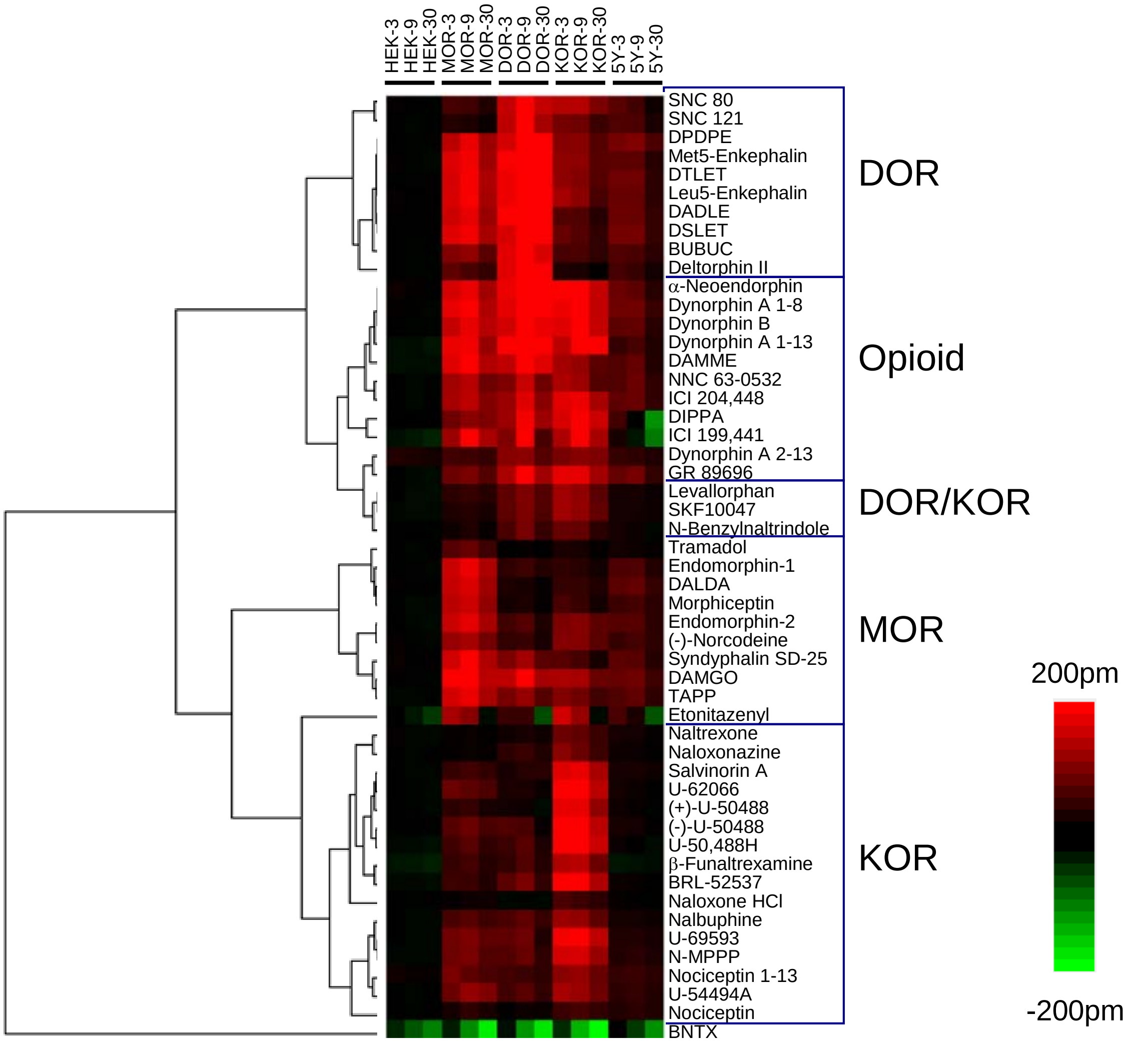

Figure S2. A false colored heat map based on the DMR of opioid ligands in five different cell lines. The cell lines were parental HEK-293, HEK-MOR, HEK-DOR, HEK-KOR and SH-SY5Y. Only ligands that resulted in a DMR with at least one response at the three time points greater than 50 pm were included in this analysis. The negative control (DMSO) was also included.

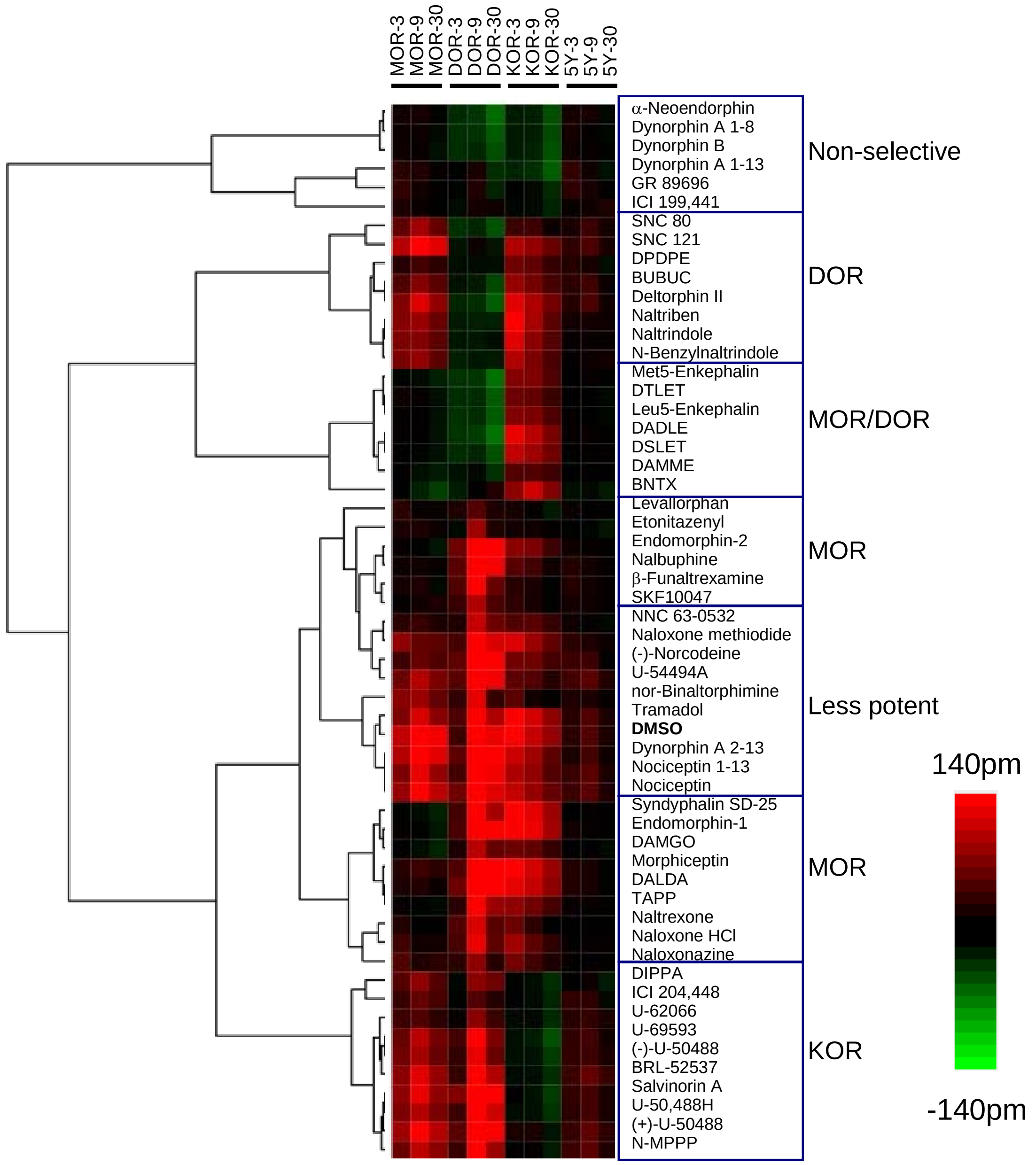

Figure S3 A false colored heat map based on the selectivity of opioid ligands to block the DMR of control agonists in respective cell lines. The DMR of a specific agonist at a fixed dose in each cell line after pretreatment with an opioid ligand in the library was used to generate the heat map. The agonists used were DAMGO (10 μM), DPDPE (10 μM), BRL-52537 (10 μM), and DAMGO (10 μM) for HEK-MOR, HEK-DOR, HEK-KOR and SH-SY5Y cells, respectively. All of the ligands in the library were assayed at 10 μM, and used to pretreat the cells for about 1 hr. All fifty-five ligands were included in the analysis. The positive controls (*i.e.*, the agonist responses in the DMSO pretreated cells) were also included.

Table S1 Opioid ligands and their affinity binding to the MOR.

Compound	Pharmacology	K_i (IC_{50}/EC_{50}) (nM)	Reference
(-)-Norcodeine	Opioid agonist	266.9	Lotsch et al., 2006
(-)-U-50488 HCl	Kappa agonist	830	Tam, 1985
(+)-U-50488 HCl	Kappa partial agonist		
SKF10047	Opioid agonist/antagonist	1900	Tam, 1985
DADLE	Opioid agonist	150	Tam, 1985
DAMME	Mu agonist	0.33	Leslie et al., 1982
DAMGO	Mu agonist	1.1	Amiche et al., 1989
DALDA	Mu agonist	1.69	Schiller et al., 1989
BUBUC	Delta agonist	2980	Gacel et al., 1990
DSLET	Delta agonist	120	Creese et al., 1976
DTLET	Delta agonist		
(Leu5)-Enkephalin	Mu agonist	7.4	Childers et al., 1979
(Met5)-Enkephalin	Mu agonist	3	Childers et al., 1979
TAPP	Mu agonist		
DIPPA	Kappa antagonist	1799 (IC_{50})	Chang et al., 1994
α-Neoendorphin	Kappa1 agonist		
β-Funaltrexamine HCl	Mu antagonist	0.33	Raynor et al., 1994
BNTX maleate	Delta antagonist	18	Reisine, 1995
BRL-52537	Kappa agonist	1560	Chen et al., 2004
DAMGO	Mu agonist	1.1	Amiche et al., 1989
Deltorphin II	Delta agonist	>1000	Reisine, 1995
DPDPE	Delta agonist	600	Amiche et al., 1989
Dynorphin A (1-13)	Kappa agonist	31	Tam, 1985
Dynorphin A (1-8)	Kappa agonist	22.3	Merg et al., 2006
Dynorphin A (2-13)	Kappa agonist		
Dynorphin B	Kappa agonist	13	Merg et al., 2006
Endomorphin-1	Mu agonist	0.67	Goldberg et al., 1998
Endomorphin-2	Mu agonist	0.43	Goldberg et al., 1998
Etonitazenyl isothiocyanate	Mu antagonist	71	Rios and Tephly, 2002
GR 89696 fumarate	Kappa agonist		
ICI 199,441 HCl	Kappa agonist	4500	Costello et al. 1988
ICI 204,448 HCl	Kappa agonist	>1000	Raynor et al., 1994
Levallorphan tartrate	Opioid antagonist	1.7	Carroll et al., 1988
Nalbuphine HCl	Mu opioid antagonist/kappa agonist	11	Raynor et al., 1994
Naloxonazine 2HCl	Opioid antagonist	0.054	Raynor et al., 1994

Naloxone HCl	Opioid antagonist	2.4	Titeler et al., 1989
Naloxone methiodide	Opioid antagonist	28.9	Lewanowitsch and Irvine, 2003
Naltrexone HCl	Opioid antagonist	0.77	Carroll et al., 1988
Naltriben mesylate	Delta antagonist	80.8	Raynor et al., 1994
Naltrindole HCl	Delta agonist	64	Reisine, 1995
N-Benzylnaltrindole HCl	Delta antagonist	11.2 (IC_{50} Ratio)**	Korlipara et al., 1994
N-MPPP*	Kappa agonist	>1000	Weerawarna et al., 1994
NNC 63-0532	ORL1 agonist	140	Titeler et al., 1989
Nociceptin	ORL1 agonist	270	Halab et al., 2002
Nociceptin (1-13) NH2	ORL1 agonist		
nor-Binaltorphimine 2HCl	Kappa antagonist	2.2	Raynor et al., 1994
Salvinorin A	Kappa agonist	1728 (EC_{50})	Rothman et al., 2007
SNC 121	Delta agonist		
SNC 80	Delta agonist		
Syndyphalin SD-25	Mu agonist	0.29 (IC_{50})	Quirion et al., 1982
Tramadol HCl	Mu agonist	2400	Gillen et al., 2000
U-50,488H mesylate	Kappa agonist		
U-54494A HCl	Kappa agonist		
U-62066	Kappa agonist	252	Clark et al., 1988
U-69593	Kappa agonist	1600	Titeler et al., 1989

* N-MPPP, N-Methyl-N-[(1S)-1-phenyl-2-(1-pyrrolidinyl)ethyl]phenylacetamide HCl.

** * IC_{50} ratio is the IC_{50} of agonist in the presence of 100nM antagonist.

Table S2 Opioid ligands and their affinity binding to the DOR

**Compound**	**Pharmacology**	**K_i (IC_{50}) (nM)**	**Reference**
(-)-Norcodeine	Opioid agonist		
(-)-U-50488 HCl	Kappa agonist	2300	Chang, 2003
(+)-U-50488 HCl	Kappa partial agonist		
SKF10047	Opioid agonist/antagonist	19000	Tam, 1985
DADLE	Opioid agonist	0.63	Chavkin, 1982
DAMME	Mu agonist	7.7 (IC_{50})	Leslie et al., 1982
DAMGO	Mu agonist	64.7	Amiche et al., 1989
DALDA	Mu agonist	19200	Schiller et al., 1989
BUBUC	Delta agonist	2.9	Gacel et al., 1990
DSLET	Delta agonist	4.8	Clark et al., 1988
DTLET	Delta agonist	22	Wang et al., 1991
(Leu5)-Enkephalin	Mu agonist	6.2	Childers et al., 1979
(Met5)-Enkephalin	Mu agonist	2	Childers et al., 1979
TAPP	Mu agonist	695	Charpentier et al., 1991
DIPPA	Kappa antagonist	>1000 (IC_{50})	Chang et al., 1994
α-Neoendorphin	Kappa1 agonist	1.17	Mansour et al., 1995
β-Funaltrexamine HCl	Mu antagonist	48	Raynor et al., 1994
BNTX maleate	Delta antagonist	0.66	Reisine, 1995
BRL-52537	Kappa agonist		
DAMGO	Mu agonist	>1000	Reisine, 1995
Deltorphin II	Delta agonist	3.3	Reisine, 1995
DPDPE	Delta agonist	2.15	Amiche et al., 1989
Dynorphin A (1-13)	Kappa agonist	>1000	Reisine, 1995
Dynorphin A (1-8)	Kappa agonist	32.6	Merg et al., 2006
Dynorphin A (2-13)	Kappa agonist		
Dynorphin B	Kappa agonist	12.8	Merg et al., 2006
Endomorphin-1	Mu agonist	>500	Goldberg et al., 1998
Endomorphin-2	Mu agonist	>500	Goldberg et al., 1998
Etonitazenyl isothiocyanate	Mu antagonist	>1000	Fichna et al., 2008
GR 89696 fumarate	Kappa agonist		
ICI 199,441 HCl	Kappa agonist		
ICI 204,448 HCl	Kappa agonist	>1000	Raynor et al., 1994
Levallorphan tartrate	Opioid antagonist	1	Childers et al., 1979
Nalbuphine HCl	Mu antagonist/kappa agonist	163	Tam, 1985
Naloxonazine 2HCl	Opioid antagonist	8.6	Raynor et al., 1994
Naloxone HCl	Opioid antagonist	16	Tam, 1985

Naloxone methiodide	Opioid antagonist	203.5	Lewanowitsch and Irvine, 2003
Naltrexone HCl	Opioid antagonist	149	Reisine, 1995
Naltriben mesylate	Delta antagonist	0.4	Spetea et al., 1998
Naltrindole HCl	Delta agonist	0.02	Reisine, 1995
N-Benzylnaltrindole HCl	Delta antagonist	459 (IC_{50} Ratio**)	Korlipara et al., 1994
N-MPPP*	Kappa agonist	>1000 (IC_{50})	Weerawarna et al., 1994
NNC 63-0532	ORL1 agonist		
Nociceptin	ORL1 agonist	>1000	Halab et al., 2002
Nociceptin (1-13) NH2	ORL1 agonist		
nor-Binaltorphimine 2HCl	Kappa antagonist	65	Raynor et al., 1994
Salvinorin A	Kappa agonist	>1000	Roth et al., 2002
SNC 121	Delta agonist	3.8	Ni et al., 1994
SNC 80	Delta agonist	1.8	Bryans, 1999
Syndyphalin SD-25	Mu agonist	1250 (IC_{50})	Quirion et al., 1982
Tramadol HCl	Mu agonist	57700	Codd et al., 1995
U-50,488H mesylate	Kappa agonist	2100	Tam, 1985
U-54494A HCl	Kappa agonist		
U-62066	Kappa agonist	9400	Wadenberg, 2003
U-69593	Kappa agonist	13400	Clark et al., 1988

* N-MPPP, N-Methyl-N-[(1S)-1-phenyl-2-(1-pyrrolidinyl)ethyl]phenylacetamide HCl.

** * IC_{50} ratio is the IC_{50} of agonist in the presence of 100nM antagonist.

Table S3 Opioid ligands and their affinity binding to the KOR

**Compound**	**Pharmacology**	**K_i (IC_{50}/EC_{50}) (nM)**	**References**
(-)-Norcodeine	Opioid agonist		
(-)-U-50488 HCl	Kappa agonist	4.2	Lahti et al., 1985
(+)-U-50488 HCl	Kappa partial agonist		
SKF10047	Opioid agonist/antagonist	1600	Tam, 1985
DADLE	Opioid agonist	1900	Lahti et al., 1985
DAMME	Mu agonist	280	Chang, 2003
DAMGO	Mu agonist	>20000	Amiche et al., 1989
DALDA	Mu agonist	26	Zhao et al., 2003
BUBUC	Delta agonist	1900	Gacel et al., 1990
DSLET	Delta agonist	>1000	Reisine, 1995
DTLET	Delta agonist	> 10000	Besse et al., 1990
(Leu5)-Enkephalin	Mu agonist	9.4	Childers et al., 1979
(Met5)-Enkephalin	Mu agonist	2.9	Childers et al., 1979
TAPP	Mu agonist		
DIPPA	Kappa antagonist	2.21 (IC_{50})	Chang et al., 1994
α-Neoendorphin	Kappa1 agonist	1.4	Meng et al., 1993
β-Funaltrexamine HCl	Mu antagonist	2.8	Raynor et al., 1994
BNTX maleate	Delta antagonist	55	Reisine, 1995
BRL-52537	Kappa agonist	0.24	Chen et al., 2004
DAMGO	Mu agonist	>20000	Reisine, 1995
Deltorphin II	Delta agonist	>1000	Reisine, 1995
DPDPE	Delta agonist	12000	Mosberg et al., 1987
Dynorphin A (1-13)	Kappa agonist	0.98	Lahti et al., 1985
Dynorphin A (1-8)	Kappa agonist	0.275	Merg et al., 2006
Dynorphin A (2-13)	Kappa agonist	680	Meng et al., 1993
Dynorphin B	Kappa agonist	3.8	Merg et al., 2006
Endomorphin-1	Mu agonist	>500	Goldberg et al., 1998
Endomorphin-2	Mu agonist	>500	Goldberg et al., 1998
Etonitazenyl isothiocyanate	Mu antagonist		
GR 89696 fumarate	Kappa agonist	41.7	Caudle et al., 1997
ICI 199,441 HCl	Kappa agonist	0.054	Kumar et al., 2000
ICI 204,448 HCl	Kappa agonist	2.69	Kumar et al., 2005
Levallorphan tartrate	Opioid antagonist	0.3	Childers et al., 1979
Nalbuphine HCl	Mu opioid antagonist/kappa agonist	61	Raynor et al., 1994
Naloxonazine 2HCl	Opioid antagonist	11	Raynor et al., 1994

Naloxone HCl	Opioid antagonist	6.3	Lahti et al., 1985
Naloxone methiodide	Opioid antagonist	1010	Lewanowitsch and Irvine, 2003
Naltrexone HCl	Opioid antagonist	0.83	Smith, 1989
Naltriben mesylate	Delta antagonist	>10000	Spetea et al., 1998
Naltrindole HCl*	Delta agonist	66	Reisine, 1995
N-Benzylnaltrindole HCl	Delta antagonist	1.3 (IC_{50} Ratio)**	Korlipara et al., 1994
N-MPPP*	Kappa agonist	1.4 (IC_{50})	Weerawarna et al., 1994
NNC 63-0532	ORL1 agonist	405	Thomsen and Hohlweg, 2000
Nociceptin	ORL1 agonist	>1000	Halab et al., 2002
Nociceptin (1-13) NH2	ORL1 agonist		
nor-Binaltorphimine 2HCl	Kappa antagonist	0.027	Raynor et al., 1994
Salvinorin A	Kappa agonist	0.63nM (EC_{50})	Chavkin et al., 2004
SNC 121	Delta agonist		
SNC 80	Delta agonist	2900	Chang, 2003
Syndyphalin SD-25	Mu agonist	13000 (IC_{50})	Quirion et al., 1982
Tramadol HCl	Mu agonist	42700	Codd et al., 1995
U-50,488H mesylate	Kappa agonist	7	Gairin et al., 1985
U-54494A HCl	Kappa agonist	21	Vonvoigtlander et al., 1987
U-62066	Kappa agonist	1.4	Lahti et al., 1985
U-69593	Kappa agonist	5.4	Lahti et al., 1985

* N-MPPP, N-Methyl-N-[(1S)-1-phenyl-2-(1-pyrrolidinyl)ethyl]phenylacetamide HCl.

** * IC_{50} ratio is the IC_{50} of agonist in the presence of 100nM antagonist.

References

1. Amiche M, Sagan S, Mor A, Delfour A and Nicholas P (1989) Dermenkephalin (Tyr-D-Met-Phe-His-Leu-Met-Asp-NH2): A potent and fully specific agonist for the delta opioid receptor. *Mol. Pharmacol.* **35**: 774-779.
2. Besse D, Lombard MC, Zajac JM, Roques and JM Besson (1990) Pre- and postsynaptic distribution of μ, δ, and κ opioid receptors in the superficial layers of the cervical dorsal horn of the rat spinal cord. *Brain Res.* **521**:15-22.
3. Bryans JS (1999) Novel Treatments for pain. *IDrugs* **2**:1170-1182

4. Carroll JA, Shaw JS and Wickenden AD (1988) The physiological relevance of low agonist affinity binding at opioid μ-receptors. *Br. J. Pharmacol.* **94**: 625-631.
5. Caudle RM, Mannes AJ and Iadarola MJ (1997) GR89,696 is a kappa-2 opioid receptor agonist and a kappa-1 opioid receptor antagonist in the guinea pig hippocampus. *PNAS USA* **283**:1342-1349.
6. Chang AC, Takemori AE and Portoghese PS (1994) 2-(3,4-Dichlorophenyl)-N-methyl-N-[(1S)-1-(3-isothiocyanatophenyl)-2-(1- pyrrolidinyl)ethyl]acetamide: An opioid receptor affinity label that produces selective and long-lasting kappa antagonism in Mice. *J. Med. Chem.* **37**:1547–1549.
7. Chang KJ (2003) The delta receptor. Informa Healthcare: New York.
8. Charpentier S, Sagan S, Delfour A and Nicolas P (1991) Dermenkephalin and deltorphin I reveal similarities within ligand-binding domains of μ- and δ-opioid receptors and an additional address subsite on the δ-receptor. *Biochem. Biophys. Res. Comm.* **179**:1161-1168.
9. Chavkin C (1982) Dynorphin is a specific endogenous ligand of the kappa opioid receptor. *Science* **215**: 413-415.
10. Chavkin C, Sud S, Jin W, Stewart J and Zjawiony JK (2004) Salvinorin A, an active component of the hallucinogenic sage salvia divinorum is a highly efficacious κ-opioid receptor agonist: structural and functional considerations. *PNAS. USA* **308**:1197-1203.
11. Chen TY, Goyagi T, Toung TJ, Kirsch JR, Hurn PD, Koehler RC and Bharkwaj A (2004) Prolonged opportunity for ischemic neuroprotection with selective kappa-opioid receptor agonist in rats. *Stroke* **35**:1180.
12. Childers SR, Creese I, Chowman AM and Snyder SH (1979) Opiate receptor binding affected differentially by opiates and opioid peptides. *Eur. J. Pharmacol.* **55**: 11-18
13. Clark MJ, Carter BD and Medzihradsky F (1988) Selectivity of ligand binding to opioid receptors in brain membranes from the rat, monkey and guinea pig. *Eur. J. Pharmacol*. **148**: 343-351.
14. Codd EE, Shank RP, Schupsky JJ and Raffe RB (1995) Serotonin and norepinephrine uptake inhibiting activity of centrally acting analgesics: structural determinants and role in antinociception. *J.Pharmacol. Exp. Ther.* **273**:1263-1270.

15. Costello GF, Main BG, Barlow JJ, Carrol JA and Shaw JS (1988) A novel series of potent and selective agonists at the opioid kappa-receptor. *Eur. J. Pharmacol.* **151**: 475-478.

16. Creese I, Feinberg AP and Snyder SH (1976) Butyrophenone influences on the opiate receptor. *Eur. J. Pharmacol.* **36**: 231-235.

17. Fichna J, Do-Rego JC, Janecki T, Staniszewska R, Poels J, Broeck JV, Costentin J, Scholler PW and Janecka A (2008) Novel highly potent μ-opioid receptor antagonist based on endomorphin-2 structure. *Bioorg Med Chem Lett* **18**:1350-1353.

18. Gacel GA, Fellion E, Baamonde A, Dauge V and Roquies BP (1990) Synthesis, biochemical and pharmacological properties of BUBUC, a highly selective and systemically active agonist for in vivo studies of δ-opioid receptors. *Peptides* **11**:983-988.

19. Gairin JE, Gouarderes C, Mazarguil H, Alvinerie P and Cros J (1985) [D-Pro10]dynorphin-(1-11) is a highly potent and selective ligand for kappa opioid receptors. *Eur. J. Pharmacol.* **106**: 457-458.

20. Gillen C, Haurand M, Kobelt DJ and Wnendt S (2000) Affinity, potency and efficacy of tramadol and its metabolites at the cloned human μ-opioid receptor. *Naunyn-schmiedeberg's Arch. Pharmacol.* **362**:116-121.

21. Goldberg IE, Rossi GC, Letchworth SR, Mathis JP, Ryan-Moro J, et al. (1998) Pharmacological characterization of endomorphin-1 and endomorphin-2 in mouse brain. *JPET* **286**:1007-1013.

22. Halab L, Becker JA, Darula Z, Tourwe D, Kieffer BL Simonin F and Lubell WD (2002) Probing opioid receptor interactions with azacycloalkane amino acids. Synthesis of a potent and selective ORL1 antagonist. *J. Med. Chem.* **45**:5353-7.

23. Koch G,Wiedermann K and Teschemacher H. (1985) Opioid activities of human β-casomorphins. *Naunyn-Schmiederberg's Archives Pharm.* **331**:351-354.

24. Korlipara VL, Takemori AE and Portochese PS (1994) N-Benzylnaltrindoles as long acting δ-opioid receptor antagonists. *J. Med. Chem.* **37**:1882.

25. Kumar V, Guo D, Cassel JA, Daubert JD, DeHaven RN, DeHaven-Hudkins DL, Gauntner EK, Gottshall SL, Greiner SL, Koblish M et al.. (2005) Synthesis and

evaluation of novel peripherally restricted κ-opioid receptor agonists. *Bioorg Med Chem Letters* **15**:1091-1095.

26. Kumar V, Marella MA, Cortes-Burgos LC, Chang AC, Cassel JA, Daubert JD, DeHaven RN, DeHaven-Hudkins DL, Gottshall SL, Mansson E et al. (2000) Arylacetamides as peripherally restricted kappa opioid receptor agonists. *Bioorg Med Chem Letters* **10**:2567-2570.
27. Lahti RA, Mickelson MM, McCall JM and VonVoigtlander PF (1985) [3H]U-69593 a highly selective ligand for the opioid kappa receptor. *Eur. J. Pharmacol.* **109**: 281-284.
28. Leslie FM, Tso A and Hurlbut DE (1982) Differential appearance of opiate receptor subtypes in neonatal rat brain. *Life Sciences* **31**:1393-1396.
29. Leslie FM, Tso A, and Hurlbut DE (1982) Differential appearance of opiate receptor subtypes in neonatal rat brain. *Life Sciences* **31**:1393-1396.
30. Lewanowitsch T and Irvine RJ (2003) Naloxone and its quaternary derivative, naloxone methiodide, have differing affinities for μ, δ, and κ opioid receptors in mouse brain homogenates. *Brain Res*. **964**:302-305.
31. Lotsch J, Skarke C, Schmidt H, Rohrbacher M, Hofmann U, Schwab M and Geisslinger G.(2006) Evidence for morphine-independent central nervous opioid effects after administration of codeine: Contribution of other codeine metabolites. *Pharmacodynamics Drug Action* **79**:35-48.
32. Mansour A, Hoversten MT, Taylor LP, Watson SJ and Akil H (1995) The cloned μ, δ, and κ receptors and their endogenous ligands: Evidence for two opioid peptide recognition cores. *Brain Res.* **700**: 89-98.
33. Meng F, Xie GX, Thompson RC, Mansour A, Goldstein A, Watson SJ and Akil H. (1993) Cloning and pharmacological characterization of a rat kappa opioid receptor. *PNAS USA* **90**:9954-9958.
34. Merg F, Filliol D, Usynin I, Bazv I, Bark N, Hurd Yl, Yakovleva T, Kieffer BL and Bakalkin G (2006) Big dynorphin as a putative endogenous ligand for the κ-opioid receptor. *J Neurochem* **97**: 292-301.
35. Mosberg HI, Omnaas JR and Goldstein A (1987) Structural requirements for delta opioid receptor binding. *Mol. Pharmacol.* **31**: 599-602.

36. Ni Q, Xu H, Partilla JS, Porreca F, Calderon SN, Rice KC, McNutt RW and Rothman RB. (1994) [3H]SNC121: A novel high affinity ligand for rat brain delta receptors: Preliminary studies. *Regulatory Peptides* **54**: 209-210.
37. Quirion R, Kiso Y and Pert CB (1982) Syndyphalin SD-25: a highly selective ligand for mu opiate receptors. *FEBS Lett.* **141**:203-206.
38. Raynor K, Kong H, Yasuda K, Yu L, Bell GI and Reisine T (1994) Pharmacological characterization of the cloned kappa-, delta-, and mu-opioid receptors. *Mol. Pharmacol.* **45**:330-334.
39. Reisine T (1995) Opiate receptors. *Neuropharm* **34**:463-472.
40. Rios GR and Tephly TR (2002) Inhbition and active sites of UDP-glocuronosyltransferases 2B7 and 1A1. *Drug Metab Dispos.* **30**: 1364-1367
41. Roth BL, Baner K, Westkaemper R, Siebert D, Rice KC, Steinburg S, Ernsberger P and Rothman RB (2002) Salvinorin A: A potent naturally occurring nonnitrogenous κ opioid selective agonist. *PNAS USA* **99**: 11934–11939.
42. Rothman RB, Murphy DL, Xu H, Godin JA, Dersch CM, Partilla JS, Tidgewell K, Schmidt and Prisinzano TE (2007) Salvinorin A: allosteric interactions at the μ-opioid receptor. *J.Pharmacol. Exp. Ther.* **320**:801-810.
43. Schiller PW, Nguyen TM, Chung NN and Lemieus C (1989) Dermorphin analogs carrying an increased positive net charge in their "message" domain display extremely high mu-opioid receptor selectivity. *J. Med. Chem.* **32**:698-703
44. Smith JA, Hunter JC, Hill RG and Hughes J (1989) A kinetic analysis of kappa-opioid agonist binding using the selective radioligand [3H]U69593. *J. Neurochem.* **53**: 27-36.
45. Spetea M, Otvos F, Toth G, Nguyen TMD, Schiller PW, Vogel Z, Borsodi A (1998) Interaction of agonist peptide [3H]Tyr-D-Ala-Phe-Phe-NH2 with μ-opioid receptor in rat brain and CHO-μ1 cell line. *Peptides* **19**: 1091-1098.
46. Tam SW (1985) (+)-[3H]SKF 10,047, (+)-[3H]ethylketocyclazocine, μ, κ, δ and phencyclidine binding sites in guinea pig brain membranes. *Eur. J. Pharmacol.* **109**: 33-41.

47. Thomsen C and Hohlweg R (2000) (8-Naphthalen-1-ylmethyl-4-oxo-1-phenyl-1,3,8-triaza-spiro[4.5]-dec-3-yl)-acetic acid methyl ester (NNC 63-0532) is a novel potent nociceptin receptor agonist. *Br. J. Pharmacol.* **131**:903.
48. Titeler M, Lyon RA, Kuhar MJ, Frost JF, Dannais RF, Leonhardt S, Bullock A, Rydeled LT, Price DL and Struble RG (1989) μ Opiate receptors are selectively labelled by [3H]carfentanil in human and rat brain. *Eur. J. Pharmacol.* **167**: 221-228.
49. VonVoigtlander PF, Hall ED, Ochoa MC, Lewis RA and Triezenberg HJ (1987) U-54494A: A unique anticonvulsant related to kappa opioid agonists. *J.Pharmacol. Exp. Ther.* **243**:542-547.
50. Wadenberg MLG (2003) A review of the properties of spiradoline: A potent and selective κ-opioid receptor agonist. *CNS Drug Review* **9**:187-198.
51. Wang H, Pelaprat D, Roques BP, Vanhove A, Chi ZQ, and Rostene W (1991) [3H]Ohmefentanyl preferentially binds to μ-opioid receptors but also labels σ-sites in rat brain sections. *Eur. J. Pharm.* **3**:341-350.
52. Weerawarna SA, Davis RD and Nelson WL (1994) Isothiocyanate-substituted kappa-selective opioid receptor ligands derived from N-methyl-N-[(1S)-1-phenyl-2-(1-pyrrolidinyl)ethyl]phenylacetamide. *J. Med. Chem.* **37**:2856-2864.
53. Zhao GM, Qian X, Schiller PW and Szeto HH (2003) Comparison of [Dmt1]DALDA and DAMGO in binding and G protein activation at μ, δ, and κ opioid receptors. *J.Pharmacol. Exp. Ther.* **307**:947-954.
